# Stereoisomeric Tris-BINOL-Menthol Bulky Monophosphites: Synthesis, Characterisation and Application in Rhodium-Catalysed Hydroformylation

**DOI:** 10.3390/molecules27061989

**Published:** 2022-03-19

**Authors:** Alexandre P. Felgueiras, Fábio M. S. Rodrigues, Rui M. B. Carrilho, Pedro F. Cruz, Vitor H. Rodrigues, Tamás Kégl, László Kollár, Mariette M. Pereira

**Affiliations:** 1 Coimbra Chemistry Centre, Department of Chemistry, University of Coimbra, Rua Larga, 3004-535 Coimbra, Portugal; alexandrefel42@gmail.com (A.P.F.); fabio.rodrigues@uc.pt (F.M.S.R.); pedro.f.cruz@fct.uc.pt (P.F.C.); 2 Centre for Physics of the University of Coimbra, Department of Physics, University of Coimbra, Rua Larga, 3004-516 Coimbra, Portugal; vhugo@fis.uc.pt; 3 Department of Inorganic Chemistry and ELKH-PTE Research Group for Selective Chemical Syntheses, University of Pécs, Ifjúság útja 6, H-7624 Pécs, Hungary; tkegl@gamma.ttk.pte.hu (T.K.); kollar@gamma.ttk.pte.hu (L.K.)

**Keywords:** BINOL, menthol, X-ray diffraction, monophosphite synthesis, Mitsunobu reaction, DFT computational methods, Tolman’s cone angle, computed electronic parameter (CEP), rhodium-catalysed hydroformylation

## Abstract

Four stereoisomeric monoether derivatives, based on axially chiral (*R*)- or (*S*)-BINOL bearing a chiral (+)- or (−)-neomenthyloxy group were synthesised and fully characterised by NMR spectroscopy and X-ray crystallography. The respective tris-monophosphites were thereof prepared and fully characterised. The coordination ability of the new bulky phosphites with Rh(CO)_2_(acac), was attested by ^31^P NMR, which presented a doublet in the range of δ = 120 ppm, with a ^1^*J*(^103^Rh-^31^P) coupling constant of 290 Hz. The new tris-binaphthyl phosphite ligands were further characterised by DFT computational methods, which allowed us to calculate an electronic (CEP) parameter of 2083.2 cm^−1^ and an extremely large cone angle of 345°, decreasing to 265° upon coordination with a metal atom. Furthermore, the monophosphites were applied as ligands in rhodium-catalysed hydroformylation of styrene, leading to complete conversions in 4 h, 100% chemoselectivity for aldehydes and up to 98% *iso*-regioselectivity. The Rh(I)/phosphite catalytic system was also highly active and selective in the hydroformylation of disubstituted olefins, including (*E*)-prop-1-en-1-ylbenzene and prop-1-en-2-ylbenzene.

## 1. Introduction

Phosphite compounds play important roles in synthetic chemistry. For instance, they can be used as antioxidants or as complex formation agents for polymers stabilisation (e.g., polyolefins, polycarbonates, acrylonitrile-butadiene-styrene polymers, polyesters, etc) [1,2]. Furthermore, phosphites have a rich coordination chemistry, being able to form complexes with most transition metals, particularly with low-valent metal complexes, due to a predominant π-back bonding [3]. Therefore, they are widely used as outstanding ligands in catalysis [3,4], namely in olefin hydroformylation reactions [5,6,7,8,9,10,11,12,13]. In particular, rhodium(I) complexes modified with bulky aryl monophosphite ligands were found to lead to highly active, chemo- and regioselective catalysts in the hydroformylation of disubstituted and internal double bounds, under relatively mild conditions [14,15,16]. This exceptional activity results from both electronic and stereo effects: on one hand, the π-acidic character of the phosphite weakens the metal-CO bond, thereby allowing a faster CO dissociation; on the other hand, the ligand’s large cone angle allows the coordination of only one phosphite to the metal centre, even when used in large excess, which results in a low global steric hindrance around the metal centre [17,18]. Moreover, the design and synthesis of chiral phosphite ligands also play a key role in the development of asymmetric catalysis [19,20,21,22]. Among them, monophosphite ligands based on the binaphthyl backbone [23] have earned a prominent status due to their synthetic availability and efficient applications in a large number of enantioselective catalytic reactions, namely in hydrogenation [24,25,26,27,28], hydrovinylation [29,30] and allylic substitutions [28,31,32,33,34].

In our previous studies, we have developed a family of *C*_3_-symmetry binaphthyl-based monophosphites [35], whose rhodium complexes provided highly active and regioselective catalysts for the hydroformylation of aryl and alkyl olefins [36,37], including substituted and internal C=C double bonds of long-chain methyl esters, terpene and steroid derivatives. As a part of our continuous research in this field, in this paper we report the synthesis and characterisation of four stereoisomeric BINOL-menthol bulky monophosphite ligands, which have axial chirality provided by the binaphthyl backbone and central chirality provided by the menthol moiety. The Tolman’s steric (cone angles) and electronic parameters were calculated using the B97D3 density functional method. In addition, ^31^P NMR spectroscopy studies in solution were performed for Rh/phosphite complexes to assess their coordination ability. Finally, their evaluation in Rh-catalysed hydroformylation of styrene and two disubstituted aryl olefins is described.

## 2. Results and Discussion

### 2.1. Synthesis and Characterisation of Monophosphites

**Scheme 1:**
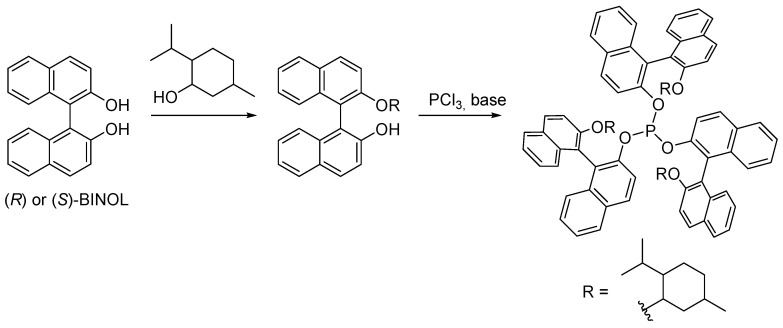
General synthetic strategy of tris-BINOL-menthol monophosphite ligands.

The synthesis of tris-BINOL-menthol-based phosphites consisted of a two-step procedure that involved the monoprotection of enantiomerically pure (*S*)- or (*R*)-BINOL with (+)- or (−)-menthol, followed by PCl_3_ phosphorylation, in the presence of a base (Scheme 1). The introduction of menthyloxy substituents at the 2-position of the 1,1’-binaphthyl backbone was intended to incorporate steric bulkiness, expecting that the three modified binaphthyloxy units at phosphorus would be “fixed” into a screw-like alignment and that this would subsequently induce helicity in order to obtain a single diastereomeric conformer for each monophosphite [27]. In addition, besides the axial chirality of (*R*)- or (*S*)-BINOL, both (+)- and (−)-menthol chiral enantiomers were used in order to obtain four different stereoisomers.

**Scheme 2:**
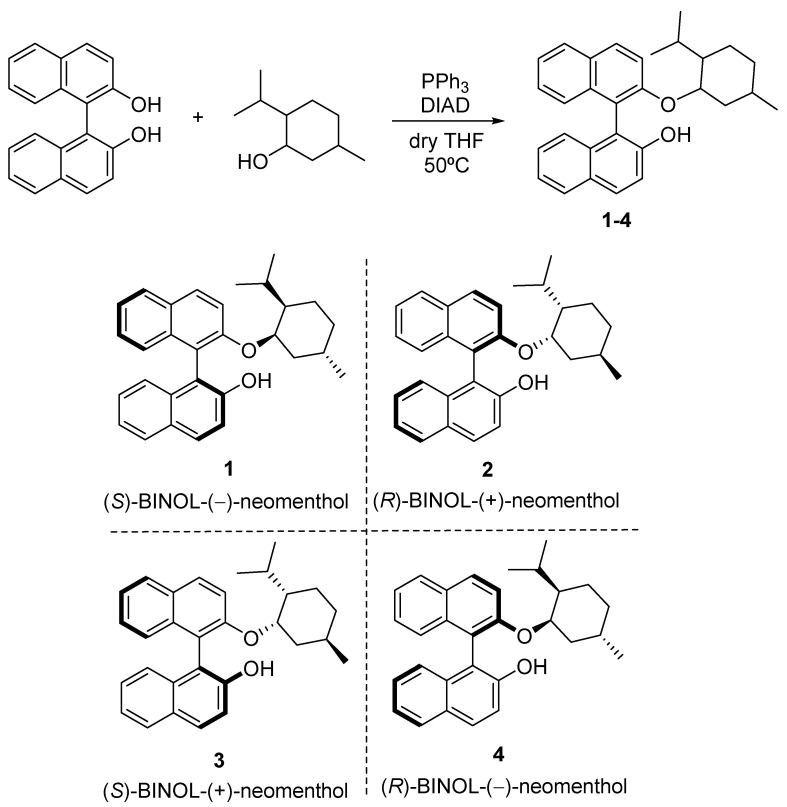
Synthesis of monoether BINOL-neomenthol stereoisomers.

The first step of the synthesis consisted of the monoetherification of axially chiral (*R*)- or (*S*)-BINOL with (+)- or (−)-menthol, in presence of triphenylphosphine and diisopropyl azodicarboxylate (DIAD), using THF as solvent (Scheme 2), following an optimised procedure of the Mitsunobu reaction [38,39]. The progress of the reaction was controlled by TLC, until full consumption of triphenylphosphine was observed. At the end, the reaction was quenched with water and successively worked up through standard procedures. Finally, the crude mixtures were purified by flash chromatography to afford the desired monoethers **1**–**4** in isolated yields of ca. 30% for all stereoisomers. It should be highlighted that, upon etherification, the absolute configurations of carbon atoms C2 and C5 in menthol fragment remained unchanged, but the absolute configuration of carbon C1 underwent inversion of configuration as a result of the Mitsunobu reaction S_N_2 mechanism [38], which resulted in the formation of BINOL-neomenthol stereoisomers.

The low reaction rate and moderate yields of the optimised etherification were attributed to the high steric hindrance of menthol, similarly to that previously observed for the Mitsunobu reaction with other secondary or tertiary alcohols [36,39]. After chromatographic purification, the unreacted BINOL was recovered and used in a subsequent monoetherification reaction. All the synthesised BINOL ether derivatives were characterised by ^1^H and ^13^C NMR spectroscopy (Figures S1–S9, Supplementary Materials), and the full assignment of the NMR signals was performed for **3** (Table S1, Supplementary Materials), using two-dimensional techniques (COSY, HMBC, HSQC) and DEPT 135 (Figures S10–S13, Supplementary Materials).

**Figure 1:**
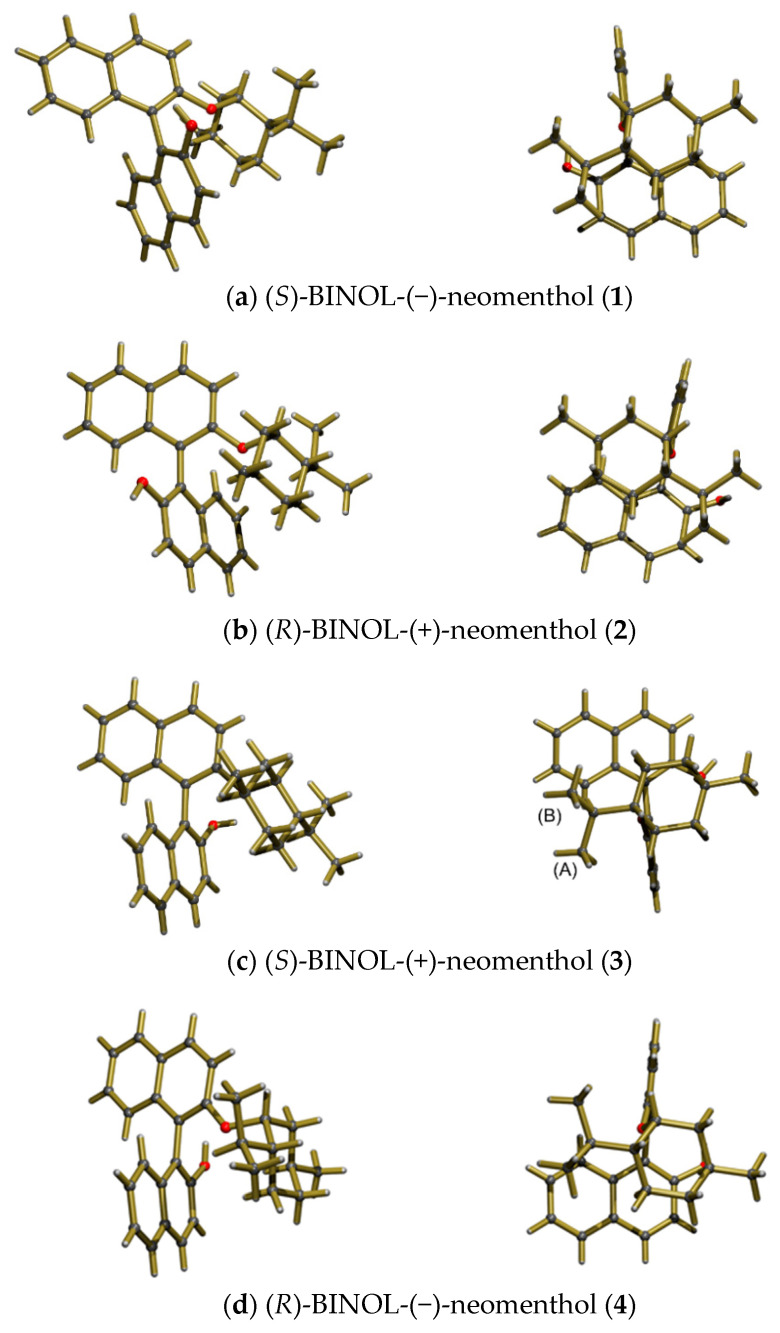
Two different perspectives of chiral moieties found in crystal structures of each stereoisomers **1**–**4**, drawn with PLATON [42] and POVRAY [43]. Coloured balls representing atoms are all of the same fixed size, thus not showing any thermal displacements.

Single-crystals of the BINOL-neomenthol stereoisomers **1**–**4** were obtained from a diethyl ether/*n*-hexane (1:1) solution (5 mg mL^−1^), by slow evaporation of the solvent at 25 °C. Then, X-ray diffraction data were collected using Mo-Kα radiation (λ = 0.71073 Å), with φ/ω scans at 296(2) K. Direct methods were applied to obtain an initial structure model which was then refined with the least-squares method. The bond lengths and angles found are in agreement with related previously reported structures [40,41]. Chirality-representative moieties found in the asymmetric units of **1**–**4** are shown in Figure 1.

**Figure 2:**
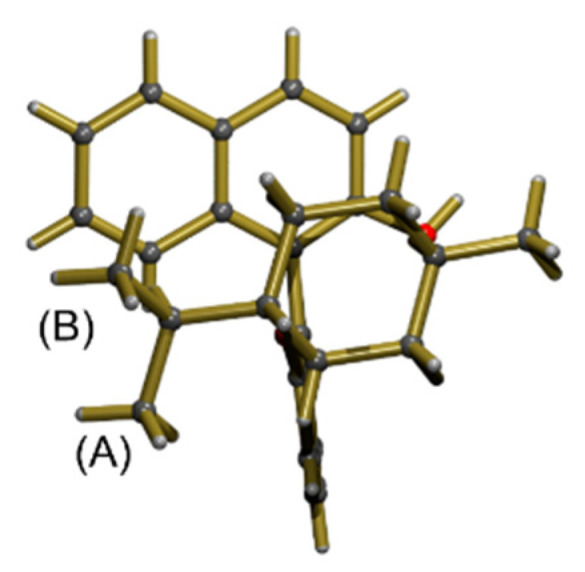
Single-crystal structure of stereoisomer **3**, showing different chemical environments of isopropyl group methyl protons (A and B).

The obtained crystal structures clearly show the structural differences between the four stereoisomers, generated by chirality of both the menthol moiety and the BINOL backbone. From the images, we can observe the same stereoconfiguration of the binaphthyl scaffold in diastereomers **1** and **3**, both synthesised from (*S*)-BINOL (Figure 1a,c). On the other hand, as expected, the crystal structures obtained for enantiomers **1** and **2** are the perfect mirror images of each other (Figure 1a,b). Furthermore, the crystal structures corroborate the differences observed in the ^1^H NMR chemical shifts of the two diastereotopic methyl groups of the isopropyl moiety for each pair of diastereomers, which result from their slightly different chemical environment due to molecular asymmetry. As illustrated in Figure 2, in stereoisomer **3**, the more shielded methyl protons B, which are above the aromatic ring current (Figure 2) give rise to a NMR signal with lower chemical shift (δ = 0.40 ppm), while the methyl group A, which is out of the shielding cone (Figure 2), produce a lower field signal with a slightly higher chemical shift (δ = 0.55 ppm).

**Scheme 3:**
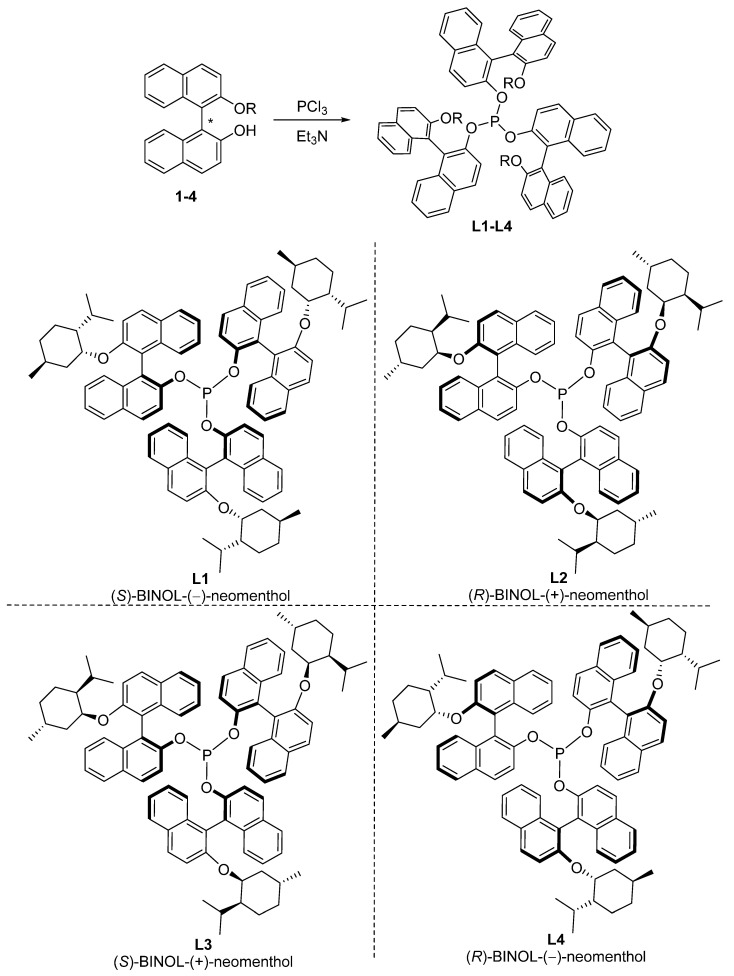
Synthesis of monophosphites **L1**–**L4**.

The second step of the monophosphite synthesis comprised the phosphorylation of monohydroxylated BINOL derivatives **1**–**4** with PCl_3_, using triethylamine (Et_3_N) simultaneously as a base and reaction solvent (Scheme 3). The reaction progress was followed by TLC analysis and ^31^P NMR spectroscopy from aliquots taken from the reaction mixture. After 5 h, the emergence of a ^31^P NMR signal at ca. δ = 136 ppm, concomitantly with the disappearance of the signal at δ = 219 ppm, confirmed the full consumption of PCl_3_ and the formation of P(OR)_3_ species. Then, the remaining Et_3_N was evaporated and the residues were purified by flash chromatography, which allowed the isolation of the desired monophosphites **L1**–**L4** in yields ranging from 65 to 68%. These were fully characterised by ^31^P, ^1^H and ^13^C NMR spectroscopy and HRMS (Figures S17–S28, Supplementary Materials).

### 2.2. NMR Studies on Complex Formation in Solution

**Figure 3:**
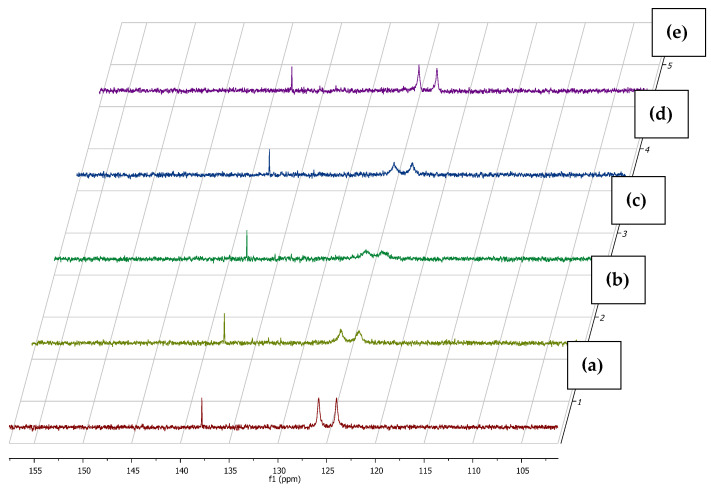
^31^P NMR spectra of **L2** + Rh(CO)_2_(acac) (molar ratio = 1:1), in toluene-d8, at different temperatures: (**a**) −3 °C; (**b**) 10 °C; (**c**) 25 °C; (**d**) 50 °C; (**e**) 80 °C (expansion of the region 105–155 ppm).

To get an insight about the coordination ability of the tris-BINOL-neomenthol monophosphite ligands, NMR studies were carried out to investigate complex formation in solution. Thus, equimolar amounts of monophosphite **L2** and Rh(CO)_2_(acac) were dissolved in toluene-d8 under an argon atmosphere at 25 °C and stirred for 1 h. Then, ^31^P NMR spectra of the mixture were acquired at variable temperatures (Figure 3b). We clearly observe a signal at *δ* = 138 ppm (*ca.* 6%, Figure S29, Supplementary Materials), assigned to the non-coordinated phosphite and the emergence of a doublet at *δ* = 122–124 ppm, with a ^1^*J*(^103^Rh-^31^P) coupling constant of ca. 290 Hz, typical of direct rhodium-phosphorus coordination [36], which confirmed the Rh-phosphite complex formation. The signal broadening, observed in the temperature range 10–50 °C, was attributed to the existence of rotational isomerism, and slow rotation in solution with consequent enhanced relaxation. At 80 °C, the ^31^P NMR shows a sharper doublet, probably due to higher motion with increasing temperatures. On the other hand, the ^31^P NMR acquired at −3 °C also presented a well-defined signal for the Rh-P complex, which was attributed to the restricted rotation at low temperature, leading to the prevalence of a single major species in solution. It should be noted that, when using a twofold excess of phosphite, no additional signals were observed in the ^31^P NMR spectra, which points towards the formation of a single Rh-P species containing only one phosphite ligand.

### 2.3. Computational Studies: Determination of Electronic and Steric Parameters

**Figure 4:**
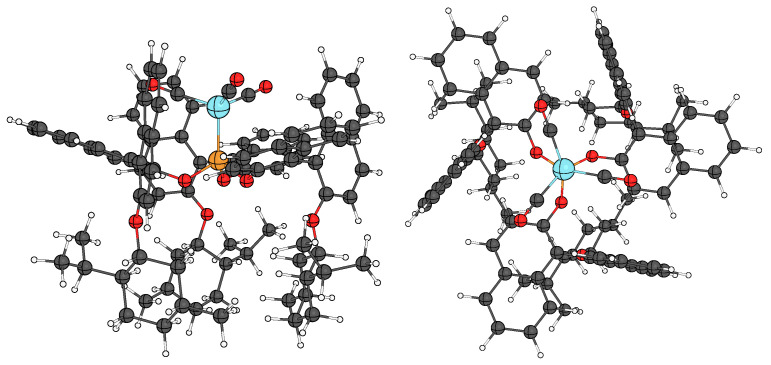
Side and top view of nickel tricarbonyl complex Ni(CO)_3_**L1**, used for the calculation of the computed steric and electronic parameters of monophosphite ligand **L1** (blue = Ni; orange = P; red: O; black = C; white = H).

The electronic (ν_A_) and steric parameters (θ) of the new tris-binaphthyl phosphite ligands were computed for ligand **L1** within the framework of the DFT methodology (see Section 3.5). The cone angles were determined, using a program developed by us, according to Tolman’s standard definition [44], in which the most stable conformer for **L1** was found to preserve the *C*_3_ symmetry. In contrast to the former alkoxy-substituted monophosphites [36], which had a cone angle in the range of 239–271 degrees, the larger steric bulkiness of the menthol substituents enforced an extremely large cone angle (345°). However, upon coordination with the metal atom, the repulsion of the spectator ligands resulted in a decrease in the cone angle, as shown for Ni(CO)_3_**L1**, whose structure was computed (Figure 4), with the cone angle decreasing to 265°. For comparison, the geometries of the rhodium(I) complexes *cis*- and *trans*-HRh(CO)_2_**L1,** considered as the hydroformylation active catalytic species, were also determined and the constrained cone angle was found to be 263°, and 264°, respectively. Therefore, the constrained cone angle showed almost negligible dependence upon the metal containing fragment.

Moreover, the computed electronic parameter (CEP), that is the total symmetric (A) carbonyl stretching frequency in the Ni(CO)_3_**L** complex type, was calculated for ligand **L1** (Figure 4). The obtained value was ν_A_ = 2083.2 cm^−1^, which is in between the CEPs of triphenyl phosphite (ν_A_ = 2085.3 cm^−1^) and trimethyl phosphite (ν_A_ = 2079.5 cm^−1^), calculated at the same level of theory [45]. Since this parameter is a generally accepted measure for the Lewis basicity of P-donor ligands, we concluded that the new tris-BINOL-neomenthol monophosphites are expected to present a stronger Lewis base character compared with that of triphenyl phosphite.

### 2.4. Evaluation in Rh-Catalysed Hydroformylation

The synthesised bulky tris-BINOL-neomenthol monophosphite ligands **L1**–**L4** were then evaluated in rhodium-catalysed hydroformylation of styrene and disubstituted styrene derivatives, such as (*E*)-prop-1-en-1-ylbenzene and prop-1-en-2-ylbenzene, using Rh(CO)_2_(acac) as a catalytic precursor in toluene. To appraise the effect of the ligand structure on the catalytic activity and selectivity, the phosphites **L1**–**L4** were first evaluated in the Rh-catalysed hydroformylation of styrene. The results are presented in Table 1.

A control experiment was carried out in the absence of phosphite ligand, at 80 °C, using a total *syngas* pressure of 10 bar (CO/H_2_ 1:1), which led to 96% conversion in 4 h, 100% chemoselectivity for aldehydes and 50% regioselectivity (Table 1, entry 1). The catalyst Rh/**L3** was then tested under the same conditions and almost full conversion (99%) was obtained in 4 h, with a higher regioselectivity (61%) for the branched aldehyde (Table 1, entry 2). When using its enantiomeric monophosphite **L4** as ligand, similar results were obtained in terms of activity, chemo- and regioselectivity (Table 1, entry 3). As observed, similar conversion was achieved with the rhodium precursor in the absence of any phosphite ligand when using 10 bar *syngas* pressure at 80 °C. However, the considerably higher regioselectivity obtained with the Rh/**L3** and Rh/**L4** catalytic systems suggests that, when bulky tris-BINOL-neomenthol phosphites are used as rhodium ligands, the hydroformylation of styrene, under these conditions, can be simultaneously catalysed either by Rh-carbonyl and Rh-phosphite species. By increasing the *syngas* pressure to 20 bar or 25 bar H_2_/CO (1:1), at the same temperature (80 °C), the catalyst Rh/**L4** also obtained full conversion in 4 h but no significant changes were observed on the reaction’s regioselectivity (Table 1, entries 4 and 5). Finally, the hydroformylation of styrene was conducted at 50 °C, either using a CO/H_2_ pressure of 20 bar (Table 1, entry 6) or 25 bar (Table 1, entries 7–9). Under these mild conditions, all catalysts Rh/**L4**, Rh/**L1** and Rh/**L2** led to close to full conversions in 4 h, with 100% chemoselectivity for aldehydes and up to 96% regioselectivity for the branched aldehyde, independently from the *syngas* pressure used. The enantiomeric excesses, determined by GC after derivatisation of the aldehyde mixtures to the corresponding carboxylic acids, were ca. 12% in all cases. Although it is well established that highly enantioselective hydroformylation reactions [46] require the use of bidentate P ligands, such as bisphosphites [47], P-chiral diphosphines [48] or hybrid phosphine-phosphite ligands [49], we hypothesised that the oxygen atom of the neomenthol ether moiety could interact with the rhodium centre through hemilabile bonds and that this could result in enantiodiscrimination. On the other hand, we also expected to observe a matching/mismatching effect of the combination of both BINOL and menthol moieties chirality on the reaction’s enantioselectivity. However, to our regret, neither of these assumptions were confirmed. Nevertheless, the high regioselectivity obtained with the new Rh/phosphite catalysts is in agreement with those previously obtained with related Rh/monodentate phosphite catalysts [36,50].

The reaction’s scope was further expanded to disubstituted aryl olefins, such as (*E*)-prop-1-en-1-ylbenzene and prop-1-en-2-ylbenzene, performed at 80 °C, using a *syngas* pressure of 25 bar (Table 2).

Remarkably, in the hydroformylation of (*E*)-prop-1-en-1-ylbenzene, the catalytic system Rh/**L2** provided 73% conversion in 4 h, along with 100% chemoselectivity for aldehydes and 80% regioselectivity for 2-phenylbutanal (Table 2, entry 1). The same catalyst was even active in the hydroformylation of less reactive 1,1-disubstituted olefin, prop-1-en-2-ylbenzene, providing 75% conversion in 18 h (Table 2, entry 2). The catalytic activity and selectivity obtained in the hydroformylation of these disubstituted olefins were within the same magnitude of those previously achieved with former C_3_-symmetry binaphthyl-based monophosphites [36]. In the latter case, it is also worth mentioning that complete chemoselectivity for aldehydes was reached, along with a substrate-controlled regioselectivity (99% to the linear aldehyde, 2-phenylbutanal) due to the preferential insertion of the carbonyl ligand at the less substituted carbon atom of the rhodium-alkyl intermediate [51].

## 3. Materials and Methods

### 3.1. Reagents and Solvents

All solvents were from commercial origin (Merck, Lisbon, Portugal) and appropriately dried by standard procedures when required [52]. Dicarbonyl(acetylacetonato)rhodium(I) was acquired from Strem Chemicals (Bischheim, France). (*R*)- and (*S*)-BINOL were purchased from RCA-Reuter Chemischer Apparatebau KG (Freiburg, Germany). The reagents diisopropylazodicarboxylate (DIAD), and (1*S*,2*R*,5*S*)-5-methyl-2-(propan-2-yl)cyclohexan-1-ol ((+)-menthol), (1*R*,2*S*,5*R*)-5-methyl-2-(propan-2-yl)cyclohexan-1-ol ((−)-menthol), triphenylphosphine, phosphorus(III) chloride, styrene, (*E*)-prop-1-en-1-ylbenzene and prop-1-en-2-ylbenzene were purchased from Merck (Lisbon, Portugal), Acros Organics (Geel, Belgium) or Alfa Aesar (Kandel, Germany). Moisture-sensitive reagents were manipulated using Schlenk techniques. The olefin substrates were passed through an alumina plug before use.

### 3.2. Instrumentation

NMR spectra were recorded on a Bruker Avance III 400 spectrometer (Wissembourg, France). The ^1^H and ^13^C chemical shifts (δ) are expressed in ppm relatively to chloroform residual peaks in CDCl_3_ (7.26 and 77.16 ppm for ^1^H and ^13^C, respectively) or to a tetramethylsilane (TMS) internal standard. For ^31^P NMR, δ are expressed relative to a phosphoric acid solution (85%) external standard. High-resolution mass spectrometry analysis was carried out on a Bruker Microtof apparatus (Billerica, MA, United States), equipped with selective ESI or MALDI detector. GC analysis was performed with an Agilent-7820A GC System (Ratingen, Germany) equipped with a non-polar capillary HP-5 column (5% diphenyl and 95% dimethylpolysiloxane), with 30 m length and 0.32 mm inside diameter equipped with an FID detector, and an Agilent-6890 (Ratingen, Germany) apparatus equipped with a chiral capillary column Supelco β-Dex 120 (20% β-cyclodextrins) with 30 m length and 0.25 mm of inside diameter, equipped with FID detector. GC-MS analysis was performed in an Agilent 7820A GC System (Ratingen, Germany), equipped with a HP-5 MS column, coupled to an Agilent 5975 MSD System Technologies spectrometer (Ratingen, Germany), using EI detector (70 eV) and helium as carrier gas. X-ray diffraction data were collected with a Bruker APEXII diffractometer (Delft, The Netherlands) (Mo-Kα radiation, λ = 0.71073 Å, graphite monochromator, using φ and ω scans at 296(2) K. Data integration and scaling were performed with SAINT [53,54], and SADABS [55] was used for empirical absorption correction. All structures were solved by direct methods using SHELXT-2014/5 [56], and full-matrix least-squares refinement on F2 of the structural model was performed by SHELXL-2016/4 [56]. All non-hydrogen atoms were refined anisotropically. Hydrogen atoms were placed at calculated idealised positions and refined as a riding model using SHELXL-2016/4 default values. The specific rotation [α] was measured in an electrical Optical Activity AA-5 polarimeter (Huntingdon, UK).

### 3.3. Monophosphite Ligands Synthesis

#### 3.3.1. Synthesis and Characterisation of BINOL Monoethers **1–4**

General procedure: To a solution of (*S*)- or (*R*)-BINOL (5.0 g, 17 mmol), dried azeotropically with toluene, (+)- or (−)-menthol (20 mmol) and triphenylphosphine (4.5 g, 17 mmol), dissolved in dry THF (100 mL), diisopropyl azodicarboxylate (DIAD) (commercial 40% solution in toluene, 7.5 mL, 17 mmol) was added dropwise, under a nitrogen atmosphere at 25 °C, and the mixture was stirred at 50 °C for 72 h. After quenching with water, the solvent was evaporated under reduced pressure and the crude mixture was dissolved in dichloromethane (50 mL). The organic layer was washed with brine (3 × 50 mL) and water (3 × 50 mL). The organic layers were then combined and dried over anhydrous Na_2_SO_4_. After solvent removal under reduced pressure, monoethers **1**–**4** were isolated by flash chromatography using silica gel as stationary phase, and CH_2_Cl_2_/*n*-hexane 1:2 as eluent.

##### (*S*)-2′-(((1*R*,2*R*,5*S*)-2-isopropyl-5-methylcyclohexyl)oxy)-[1.1′-binaphthalen]-2-ol (**1**)

Yield: 27% (2.0 g, 4.7 mmol). ^1^H NMR (400 MHz, CDCl_3_) δ/ppm: 8.00 (d, *J* = 9.1 Hz, 1H). 7.89 (d, *J* = 8.6 Hz, 2H), 7.85 (d, *J* = 8.1 Hz, 1H), 7.47 (d, *J* = 9.1 Hz, 1H), 7.38–7.35 (m, 2H), 7.30–7.24 (m, 3H), 7.18 (t, *J* = 7.6 Hz, 1H), 7.05 (d, *J* = 8.4 Hz, 1H), 5.13 (s, 1H), 4.77 (brs, 1H), 1.83 (d, *J* = 13.8 Hz, 1H), 1.55–1.46 (m, 1H), 1.34 (d, *J* = 10.4 Hz, 1H), 1.14 (d, *J* = 8.9 Hz, 1H), 0.89 (d, *J* = 6.7 Hz, 3H), 0.86–0.77 (m, 2H) 0.82 (d, *J* = 6.6 Hz, 3H), 0.64–0.65 (m, 3H), 0.30 (d, *J* = 5.2 Hz, 3H). ^13^C NMR (101 MHz, CDCl_3_) δ/ppm 154.1, 151.9, 134.6, 134.1, 130.9, 129.6, 129.4, 129.1, 128.2, 128.0, 127.2, 126.1, 125.4, 125.3, 124.1, 123.1, 117.6, 117.3, 115.8, 115.7, 75.0, 47.8, 38.2, 34.7, 29.3, 25.6, 24.5, 21.8, 21.1, 21.0. HRMS (ESI): *m*/*z* calcd. for C_30_H_33_O_2_. [M+H]^+^: 425.2475 found: 425.2482. [α]_D_^25^: +105 (c 1.0, CH_2_Cl_2_).

##### (*R*)-2′-(((1*S*,2*S*,5*R*)-2-isopropyl-5-methylcyclohexyl)oxy)-[1.1′-binaphthalen]-2-ol (**2**)

Yield: 31% (2.1 g, 5.3 mmol). ^1^H NMR (400 MHz, CDCl_3_) δ/ppm 8.00 (d, *J* = 9.1 Hz, 1H), 7.88 (d, *J* = 8.1 Hz, 1H), 7.87 (d, *J* = 8.9 Hz, 1H), 7.83 (d, *J* = 8.1 Hz, 1H), 7.46 (d, *J* = 9.1 Hz, 1H), 7.38–7.33 (m, 2H), 7.29–7.22 (m, 3H), 7.19–7.15 (m, 1H), 7.02 (d, *J* = 8.4 Hz, 1H), 5.09 (s, 1H), 4.76 (brs, 1H), 1.83–1.79 (m, 1H), 1.51–1.44 (m, 1H), 1.34–1.31 (m, 1H), 1.13–1.10 (m, 1H), 0.80 (d, *J* = 6.7 Hz, 3H), 0.85–0.75 (m, 2H), 0.81 (d, *J* = 6.7 Hz, 3H), 0.61–0.51 (m, 3H), 0.27 (d, *J* = 5.9 Hz, 3H). ^13^C NMR (101 MHz, CDCl_3_) δ/ppm 154.1, 151.9, 134.6, 134.1, 130.9, 129.6, 129.4, 129.2, 128.2, 128.0, 127.2, 126.1, 125.4, 125.3, 124.2, 123.1, 117.6, 117.3, 115.8, 115.7, 75.0, 47.8, 38.3, 34.7, 29.3, 25.7, 24.5, 21.8, 21.1, 21.0. HRMS (ESI): *m*/*z* calcd. for C_30_H_33_O_2_, [M+H]^+^: 425.2475 found: 425.2465. [α]_D_^25^: −105 (c 1.0, CH_2_Cl_2_).

##### (*S*)-2′-(((1*S*,2*S*,5*R*)-2-isopropyl-5-methylcyclohexyl)oxy)-[1.1′-binaphthalen]-2-ol (**3**) 

Yield: 32% (2.3 g, 5.3 mmol). ^1^H NMR (400 MHz, CDCl_3_) δ/ppm 7.96 (d, *J* = 9.1 Hz, 1H), 7.85 (d, *J* = 8.0 Hz, 1H), 7.84 (d, *J* = 9.1 Hz, 1H), 7.80 (d, *J* = 8.1 Hz, 1H), 7.40 (d, *J* = 9.1 Hz, 1H), 7.34–7.29 (m, 1H), 7.31 (d, *J* = 8.9 Hz, 1H), 7.25–7.22 (m, 3H), 7.17–7.12 (m, 1H), 7.02 (d, *J* = 8.4 Hz, 1H), 4.95 (s, 1H), 4.65 (brs, 1H), 2.07–2.01 (m, 1H), 1.49–1.43 (m, 1H), 1.42–1.34 (m, 1H), 1.24–1.19 (m, 1H), 0.97–0.90 (m, 1H), 0.72 (d, *J* = 6.2 Hz, 3H), 0.70–0.61 (m, 3H), 0.55 (d, *J* = 6.3 Hz, 3H), 0.51–0.44 (m, 1H), 0.39 (d, *J* = 6.4 Hz, 3H). ^13^C NMR (101 MHz, CDCl_3_) δ/ppm 154.5, 151.4, 134.5, 134.1, 130.9, 129.6, 129.1, 129.1, 128.2, 127.9, 127.3, 126.0, 125.4, 124.9, 124.0, 123.1, 117.3, 116.1, 115.7, 114.9, 74.3, 47.8, 38.7, 34.9, 28.7, 26.2, 24.6, 22.5, 20.8, 20.7. HRMS (ESI): *m*/*z* calcd. for C_30_H_32_NaO_2_, [M+Na]^+^: 447.2295 found: 447.2292. [α]_D_^25^: −45 (c 1.0, CH_2_Cl_2_).

##### (*R*)-2′-(((1*R*,2*R*,5*S*)- 2-isopropyl-5-methylcyclohexyl)oxy)-[1.1′-binaphthalen]-2-ol (**4**)

Yield: 32% (2.3 g, 5.3 mmol). ^1^H NMR (400 MHz, CDCl_3_) δ/ppm 8.01 (d, *J* = 9.1 Hz, 1H), 7.88 (d, *J* = 7.9 Hz, 1H), 7.86 (d, *J* = 8.5 Hz, 1H), 7.82 (d, *J* = 8.1 Hz, 1H), 7.43 (d, *J* = 9.1 Hz, 1H), 7.37–7.31 (m, 1H), 7.32 (d, *J* = 8.8 Hz, 1H), 7.28–7.24 (m, 3H), 7.18–7.14 (m, 1H), 7.03 (d, *J* = 8.4 Hz, 1H), 4.95 (s, 1H), 4.67 (brs, 1H), 2.09–2.05 (m, 1H), 1.50–1.46 (m, 1H), 1.43–1.33 (m, 1H), 1.26–1.20 (m, 1H) 1.00–0.93 (m, 1H), 0.77–0.69 (m, 3H), 0.75 (d, *J* = 6.6 Hz, 3H), 0.56 (d, *J* = 6.3 Hz, 3H), 0.52–0.48 (m, 1H), 0.40 (d, *J* = 6.4 Hz, 3H). ^13^C NMR (101 MHz, CDCl_3_) δ/ppm 154.5, 151.4, 134.5, 134.1, 130.9, 129.6, 129.1, 129.1, 128.2, 127.9, 127.3, 126.0, 125.4, 124.9, 124.0, 123.1, 117.3, 116.1, 115.7, 114.9, 74.3, 47.8, 38.7, 34.9, 28.7, 26.2, 24.6, 22.5, 20.8, 20.7. HRMS (ESI): *m*/*z* calcd. for C_30_H_33_O_2_. [M+H]^+^: 425.2475 found: 425.2474. [α]_D_^25^: +45 (c 1.0, CH_2_Cl_2_).

#### 3.3.2. Synthesis and Characterisation of Monophosphites **L1**–**L4**

General procedure: A dried Schlenk flask was charged with the BINOL monoether **1**–**4** (1.5 g, 3.5 mmol), which was azeotropically dried with toluene, then placed under argon atmosphere and dissolved in dry triethylamine (7 mL). The solution was cooled to 0 °C and PCl_3_ (0.1 mL, 1.1 mmol) was slowly added with stirring. After 5 h, Et_3_N was evaporated under reduced pressure. Then, the residue was dissolved in dichloromethane/*n*-hexane (1:1) and filtered through a silica plug. The monophosphite ligands **L1**–**L4** were further purified by flash chromatography using silica gel as stationary phase and dichloromethane/*n*-hexane (1:1) as eluent.

##### Tris-[(*S*)-2′-(((1*R*,2*R*,5*S*)-2-isopropyl-5-methylcyclohexyl)oxy)-[1,1′-binaphthalen]-2-yl]-phosphite (**L1**)

Yield: 64% (0.84 g, 0.64 mmol).^1^H NMR (400 MHz, CDCl_3_) δ/ppm: 7.84 (d, *J* = 9.0 Hz, 3H), 7.70 (d, *J* = 8.1 Hz, 3H), 7.53 (d, *J* = 7.9 Hz, 3H), 7.49 (d, *J* = 9.1 Hz, 3H), 7.27–7.24 (m, 3H), 7.11 (d, *J* = 8.9 Hz, 3H), 7.07–7.04 (m, 6H) 6.99–6.96 (m, 3H), 6.81 (d, *J* = 8.4 Hz, 3H), 6.48 (d, *J* = 8.9 Hz, 3H), 6.38 (d, *J* = 8.4 Hz, 3H), 4.75 (s, 3H), 1.68–1.58 (m, 6H), 1.26–1.19 (m, 6H) 1.04–1.01 (m, 3H), 0.96 (d, *J* = 6.6 Hz, 9H) 0.89–0.84 (m, 3H) 0.76 (d, *J* = 6.7 Hz, 9H), 0.70–0.64 (m, 3H), 0.58–0.42 (m, 6H), 0.16 (d, *J* = 6.4 Hz, 9H). ^13^C NMR (101 MHz, CDCl_3_) δ/ppm 153.3, 147.3, 134.4, 133.6, 130.2, 129.5, 128.6, 128.5, 127.4, 127.3, 125.9, 125.8, 125.3, 123.9, 123.5, 122.9, 121.2, 121.2, 120.4, 115.8, 74.4, 47.9, 38.2, 34.7, 28.8, 25.3, 24.1, 21.7, 21.3, 21.1. ^31^P NMR (162 MHz, CDCl_3_) δ/ppm 137.81.

##### Tris-[(*R*)-2′-(((1*S*,2*S*,5*R*)-2-isopropyl-5-methylcyclohexyl)oxy)-[1,1′-binaphthalen]-2-yl]-phosphite (**L2**)

Yield: 67% (0.88 g, 0.67 mmol). ^1^H NMR (400 MHz, CDCl_3_) δ/ppm: 7.84 (d, *J* = 9.0 Hz, 3H), 7.70 (d, *J* = 8.1 Hz, 3H), 7.52 (d, *J* = 8.0 Hz, 3H), 7.49 (d, *J* = 9.1 Hz, 3H), 7.27–7.23 (m, 3H), 7.11 (d, *J* = 8.9 Hz, 3H), 7.07–7.01 (m, 6H) 6.98–6.94 (m, 3H), 6.81 (d, *J* = 8.4 Hz, 3H), 6.48 (d, *J* = 8.9 Hz, 3H), 6.38 (d, *J* = 8.4 Hz, 3H), 4.75 (s, 3H), 1.68–1.59 (m, 6H), 1.29–1.19 (m, 6H) 1.03–1.01 (m, 3H), 0.96 (d, *J* = 6.6 Hz, 9H) 0.88–0.80 (m, 3H) 0.76 (d, *J* = 6.7 Hz, 9H), 0.70–0.64 (m, 3H), 0.55–0.41 (m, 6H), 0.16 (d, *J* = 6.4 Hz, 9H).^13^C NMR (101 MHz, CDCl_3_) δ/ppm 153.5, 147.4, 134.5, 133.8, 130.4, 129.6, 128.7, 128.6, 127.6, 127.4, 126.1, 125.9, 125.4, 124.0, 123.7, 123.0, 121.4, 121.3, 120.5, 115.9, 74.5, 48.0, 38.3, 34.8, 29.0, 25.5, 24.3, 21.8, 21.4, 21.2. ^31^P NMR (162 MHz, CDCl_3_) δ/ppm: 137.80.

##### Tris-[(*S*)-2′-(((1*S*,2*S*,5*R*)-2-isopropyl-5-methylcyclohexyl)oxy)-[1,1′-binaphthalen]-2-yl]-phosphite (**L3**)

Yield: 68% (0.89 g, 0.68 mmol). ^1^H NMR (400 MHz, CDCl_3_) δ/ppm: 7.89 (d, *J* = 9.0 Hz, 3H), 7.75 (d, *J* = 8.0 Hz, 3H), 7.49 (d, *J* = 9.1 Hz, 3H), 7.40 (d, *J* = 8.0 Hz, 3H), 7.32–7.28 (m, 3H), 7.20 (d, *J* = 8.8 Hz, 3H), 7.10–7.03 (m, 6H) 6.98–6.94 (m, 3H), 6.92 (d, *J* = 8.4 Hz, 3H), 6.42 (d, *J* = 8.4 Hz, 3H), 6.37 (d, *J* = 8.8 Hz, 3H), 4.70 (s, 3H), 2.35–2.31 (m, 3H), 1.72–1.64 (m, 3H), 1.39–1.28 (m, 6H), 1.14–1.10 (m, 3H), 1.02–0.96 (m, 3H), 0.92–0.85 (m, 3H), 0.73 (d, *J* = 6.6 Hz, 9H), 0.68–0.60 (m, 6H), 0.53 (s, 9H), 0.36 (d, *J* = 6.2 Hz, 9H). ^13^C NMR (101 MHz, CDCl_3_) δ/ppm: 152.9, 146.8, 134.4, 133.9, 130.3, 130.0, 128.8, 128.6, 127.6, 127.4, 126.2, 126.0, 125.7, 125.3, 124.2, 122.8, 120.8, 120.8, 119.7, 114.2, 73.1, 47.7, 38.7, 35.0, 28.7, 25.8, 24.5, 22.2, 20.8, 20.7. ^31^P NMR (162 MHz, CDCl_3_) δ/ppm: 135.9.

##### Tris-[(*R*)-2′-(((1*R*,2*R*,5*S*)-2-isopropyl-5-methylcyclohexyl)oxy)-[1,1′-binaphthalen]-2-yl]-phosphite (**L4**)

Yield: 65% (0.85 g, 0.65 mmol). ^1^H NMR (400 MHz, CDCl_3_) δ/ppm: 7.88 (d, *J* = 9.1 Hz, 3H), 7.74 (d, *J* = 8.1 Hz, 3H), 7.49 (d, *J* = 9.1 Hz, 3H), 7.39 (d, *J* = 8.0 Hz, 3H), 7.29–7.27 (m, 3H), 7.19 (d, *J* = 8.8 Hz, 3H), 7.09–7.01 (m, 6H), 6.97–6.93 (m, 3H), 6.91 (d, *J* = 8.5 Hz, 3H), 6.41 (d, *J* = 8.5 Hz, 3H), 6.36 (d, *J* = 8.8 Hz, 3H), 4.69 (s, 3H), 2.34–2.01 (m, 3H), 1.73–1.61 (m, 3H), 1.38–1.34 (m, 3H), 1,26 (s, 3H), 1.13–1.09 (m, 3H), 1.01–0.95 (m, 3H), 0.91–0.84 (m, 6H), 0.71(d, *J* = 6.6 Hz, 9H) 0.66–0.60 (m, 3H), 0.52 (s, 9H), 0.35 (d, *J* = 6.1 Hz, 9H). ^13^C NMR (101 MHz, CDCl_3_) δ/ppm: 152.7, 146.7, 134.2, 133.8, 130.2, 129.8, 128.5, 127.8, 127.2, 126.1, 125.6, 125.2, 124.8, 124.0, 122.7, 120.7, 119.5, 117.1, 114.8, 114.0, 73.0, 47.6, 38.6, 34.8, 28.6, 25.6, 24.3, 22.1, 20.7, 20.5. ^31^P NMR (162 MHz, CDCl_3_) δ/ppm 135.9. HRMS (MALDI-TOF): *m*/*z* calcd. for C_90_H_92_O_6_P [M−H]^+^: 1299.6626 found: 1299.6279.

### 3.4. Rh(I)/Monophosphite Complex Formation in Solution

A dried Schlenk tube was charged with Rh(CO)_2_(acac) (2.6 mg,0.01 mmol) and the monophosphite ligand **L2** (0.01 mmol), under an argon atmosphere. The solids were dissolved in CDCl_3_ (0.5 mL), and the mixture was stirred for 1 h, at 25 °C. Then, variable temperature ^31^P NMR spectra of the resulting solution were registered, ranging from −3 °C to 80 °C.

### 3.5. DFT Computational Studies

The electronic (CEP) and steric parameters (θ) were computed within the framework of the DFT methodology after obtaining its global minimum via Monte Carlo simulations using the OPLS-AA force field [57,58], within the TINKER suite of programs [59], followed by geometry optimisations at the B97D3 functional [60] in combination with the def2-SVP basis set [61], utilising the Gaussian 16 software package [62].

### 3.6. Catalytic Hydroformylation Procedure

A 60 mL autoclave was charged with the monophosphite ligand **L1**–**L4** (0.030 mmol) and the system was purged by three cycles of CO/H_2_ (1:1) and vacuum. Then, a solution of [Rh(CO)_2_(acac)] (1.5 mg, 0.006 mmol) in toluene (3 mL) was introduced via cannula, under vacuum. After 1 h incubation at 80 °C and 40 bar of *syngas*, the substrate (2.32 mmol), previously passed through an aluminium oxide (grade I) column, was dissolved in toluene (4 mL) and subsequently introduced through the inlet cannula. Then, the temperature and *syngas* pressure were set to the desired values (see Table 1 and Table 2) and the reaction was conducted under magnetic stirring for the selected time with constant *syngas* pressure. The conversion, chemo- and regioselectivity were determined by GC analysis of the reaction mixture.

## 4. Conclusions

We have efficiently synthesised four stereoisomeric BINOL-neomenthol monoethers, which were characterised by NMR and X-ray crystallography, and the corresponding tris-monophosphite ligands. Complex formation studies, performed by ^31^P NMR spectroscopy in solution, using monophosphite **L2** and Rh(CO)_2_(acac), pointed toward the formation of a single Rh(I)-carbonyl-phosphite complex, as demonstrated by a broad doublet at δ = 120 ppm, with a ^1^*J*(^103^Rh-^31^P) coupling constant of 290 Hz, typical of direct Rh-P coordination. The signal broadening was attributed to the existence of rotational isomerism in solution as demonstrated by variable temperature ^31^P NMR experiments. DFT computational studies allowed us to calculate an exceptionally large cone angle of 345° (decreasing to 265° upon coordination with a metal atom), and an electronic parameter (CEP) of 2083.2 cm^−1^, a value in between those calculated for triphenyl phosphite and trimethyl phosphite, suggesting that the BINOL-menthol monophosphites have an intermediate Lewis-base character. The monophosphites were efficiently applied as ligands in rhodium-catalysed hydroformylation of styrene and two disubstituted aryl olefins, (*E*)-prop-1-en-1-ylbenzene and prop-1-en-2-ylbenzene, leading to active, chemo- and regioselective catalytic systems. The combination of the axial chirality of the BINOL backbone with the central chirality of the menthol moiety might be promising for the development of other asymmetric catalytic reactions, where the presence of the hemilabile ether oxygen atoms and/or the matching–mismatching effect of the chiral scaffolds can be crucial to achieve high diastereo- or enantioselectivity, such as hydrogenation, allylic substitution and hydrovinylation.

## Figures and Tables

**Table 1:** Evaluation of monophosphite ligands **L1**–**L4** in Rh-catalysed hydroformylation of styrene ^a^.

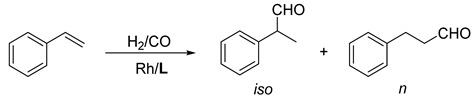					
Entry	Ligand (L)	P (bar)	T (°C)	Conversion (%)	Regio. (*iso*/*n* ratio)
1	-	10	80	96	50/50
2	**L3**-(*S*)-BINOL-(−)-menthol	10	80	99	61/39
3	**L4**-(*R*)-BINOL-(+)-menthol	10	80	99	59/41
4	**L4**-(*R*)-BINOL-(+)-menthol	20	80	99	65/35
5	**L4**-(*R*)-BINOL-(+)-menthol	25	80	99	64/36
6	**L4**-(*R*)-BINOL-(+)-menthol	20	50	92	94/6
7	**L4**-(*R*)-BINOL-(+)-menthol	25	50	95	96/4
8	**L1**-(*S*)-BINOL-(+)-menthol	25	50	93	96/4
9	**L2**-(*R*)-BINOL-(−)-menthol	25	50	94	96/4

^a^ Reaction conditions: 2.32 mmol styrene; 0.006 mmol Rh(CO)_2_(acac), 0.03 mmol phosphite ligand; styrene/Rh = 400, P/Rh = 5, t = 4 h; Chemoselectivity for aldehydes was ≥ 99% in all cases.

**Table 2:** Results of catalytic hydroformylation of (*E*)-prop-1-en-1-ylbenzene and prop-1-en-2-ylbenzene using Rh/**L2** catalyst ^a^.

Entry	Substrate	Time (h)	Conversion (%)	Major Product (Regioselectivity, %)
1	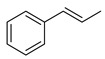	4	73	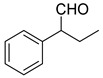 80
2	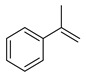	18	75	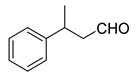 99

^a^ Reaction conditions: 2.32 mmol olefin; 0.006 mmol Rh(CO)_2_(acac), 0.03 mmol **L2**; olefin/Rh = 400, P/Rh = 5; P(CO/H_2_)(1:1) = 25 bar; T = 80 °C. Chemoselectivity for aldehydes was ≥ 99% in all cases.

## Data Availability

Crystal data of **1**-**4** are available at The Cambridge Crystallographic Data Centre (http://www.ccdc.cam.ac.uk/).

## Supplementary Materials

The supporting information can be downloaded, including NMR spectra of all new compounds and crystallographic data of compounds **1**–**4**. The following supporting information can be downloaded at: https://www.mdpi.com/article/10.3390/molecules27061989/s1, Figure S1: ^1^H NMR spectrum of (*S*)-BINOL-(–)-neomenthol (**1**) in CDCl_3_; Figure S2: ^13^C NMR spectrum of (*S*)-BINOL-(–)-neomenthol (**1**) in CDCl_3_; Figure S3: HRMS spectrum of (*S*)-BINOL-(–)-neomenthol (**1**); Figure S4: ^1^H NMR spectrum of (*R*)-BINOL-(+)-neomenthol (**2**) in CDCl_3_; Figure S5: ^13^C NMR spectrum of (*R*)-BINOL-(+)-neomenthol (**2**) in CDCl_3_; Figure S6: HRMS spectrum of (*R*)-BINOL-(+)-neomenthol (**2**); Figure S7: ^1^H NMR spectrum of (*S*)-BINOL-(+)-neomenthol (**3**) in CDCl_3_; Figure S8: ^13^C NMR spectrum of (*S*)-BINOL-(+)-neomenthol (**3**) in CDCl_3_; Figure S9: HRMS spectrum of (*S*)-BINOL-(+)-neomenthol (**3**); Figure S10: COSY spectrum of (*S*)-BINOL-(+)-neomenthol (**3**) in CDCl_3_; Figure S11: HMBC spectrum of (*S*)-BINOL-(+)-neomenthol (**3**) in CDCl_3_; Figure S12: HSQC spectrum of (*S*)-BINOL-(+)-neomenthol (**3**) in CDCl_3_; Figure S13: ^13^C NMR (1) and DEPT 135 (2) spectra of (*S*)-BINOL-(+)-neomenthol (**3**) in CDCl_3_.; Table S1: ^1^H and ^13^C NMR assignments for **3**; Figure S14: ^1^H NMR spectrum of (*R*)-BINOL-(–)-neomenthol (**4**) in CDCl_3_; Figure S15: ^1^H NMR spectrum of (*R*)-BINOL-(–)-neomenthol (**4**) in CDCl_3_; Figure S16: HRMS spectrum of (*R*)-BINOL-(–)-neomenthol (**4**); Figure S17: ^1^H NMR spectrum of **L1** in CDCl_3_; Figure S18: ^31^P NMR spectrum of **L1** in CDCl_3_; Figure S19: ^1^H NMR spectrum of **L2** in CDCl_3_; Figure S20: ^13^C NMR spectrum of **L2** in CDCl_3_; Figure S21: ^31^P NMR spectrum of **L2** in CDCl_3_; Figure S22: ^1^H NMR spectrum of **L3** in CDCl_3_; Figure S23: ^13^C NMR spectrum of **L3** in CDCl_3_; Figure S24: ^31^P NMR spectrum of **L3** in CDCl_3_; Figure S25: ^1^H NMR spectrum of **L4** in CDCl_3_; Figure S26: ^13^C NMR spectrum of **L4** in CDCl_3_; Figure S27: ^31^P NMR spectrum of **L4** in CDCl_3_; Figure S28: HRMS (MALDI-TOF) spectrum of **L4**; Table S2: Selected crystallographic data for compounds **1**-**4**.

## Sample Availability

Samples of the compounds are not available from the authors.
