# Supplementary material for: Stereoisomeric Tris-BINOL-Menthol Bulky Monophosphites: Synthesis, Characterisation and Application in Rhodium-Catalysed Hydroformylation
Source: Molecules. 2022 Mar 19;27(6):1989. doi: 10.3390/molecules27061989 (PMC8954005; doi:10.3390/molecules27061989)
Supplement: Supplementary file 1 [file molecules-27-01989-s001.zip › molecules-1600597-supplementary.pdf]

Supplementary material

for

Stereoisomeric Tris-BINOL-Menthol Bulky Monophosphites: Synthesis, Characterisation and Application in Rhodium-Catalysed Cydroformylation

Alexandre P. Felgueiras,[a,‡] Fábio M. S. Rodrigues,[a,‡] Rui M. B. Carrilho,[a,*] Pedro F. Cruz,[a] Vitor H. Rodrigues,[b] Tamás Kégl,[c] Lászlo Kollár[c] and Mariette M. Pereira [a,*]

[a] *Coimbra Chemistry Centre, Department of Chemistry, University of Coimbra, Rua Larga, 3004-535, Coimbra, Portugal*

[b] *Centre for Physics of the University of Coimbra, Department of Physics, University of Coimbra, Portugal*

[c] *Department of Inorganic Chemistry and ELKH-PTE Research Group for Selective Chemical Syntheses, University of Pécs, H-7622 Pécs, Hungary*

Index

1. NMR and HRMS spectra **S2**

2. Crystallographic data **S18**

1. NMR and HRMS spectra

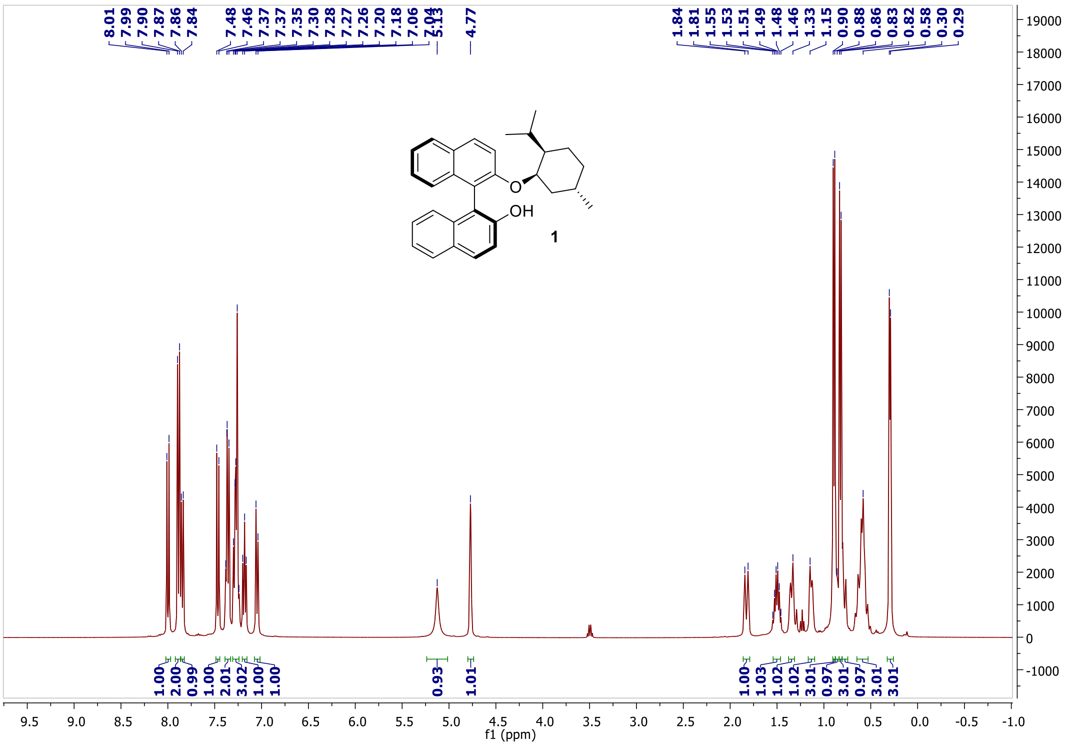

Figure S1. ^{1}H NMR spectrum of (*S*)-BINOL-(−)-neomenthol (**1**) in $CDCl_3$.

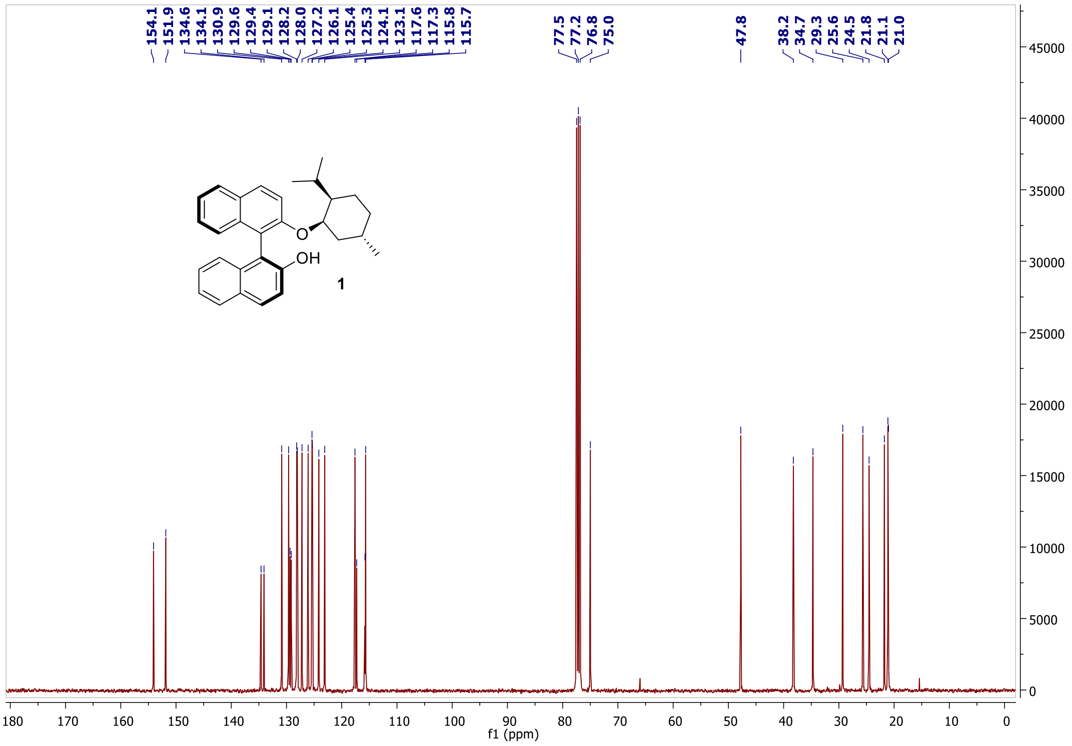

Figure S2. ^{13}C NMR spectrum of (*S*)-BINOL-(−)-neomenthol (**1**) in $CDCl_3$.

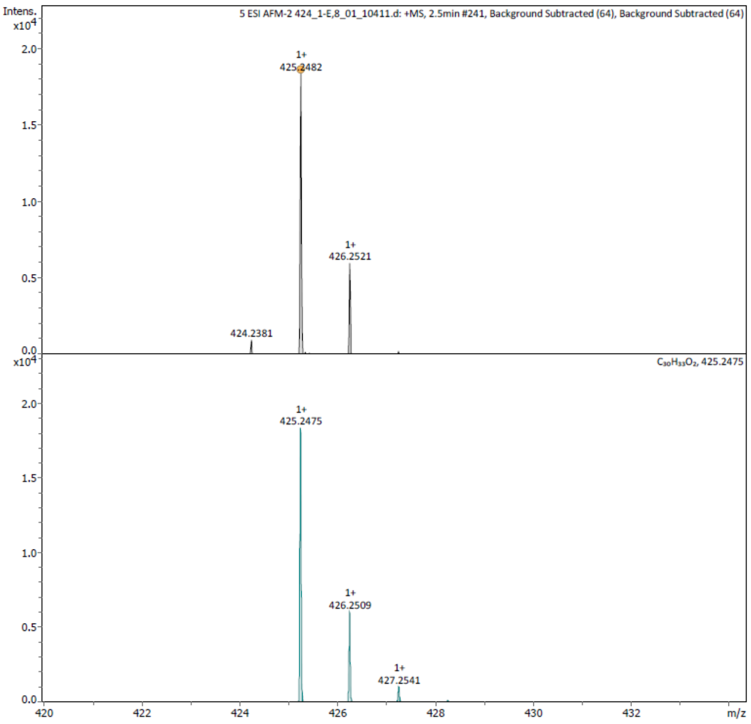

Figure S3. HRMS spectrum of (*S*)-BINOL-(−)-neomenthol (**1**).

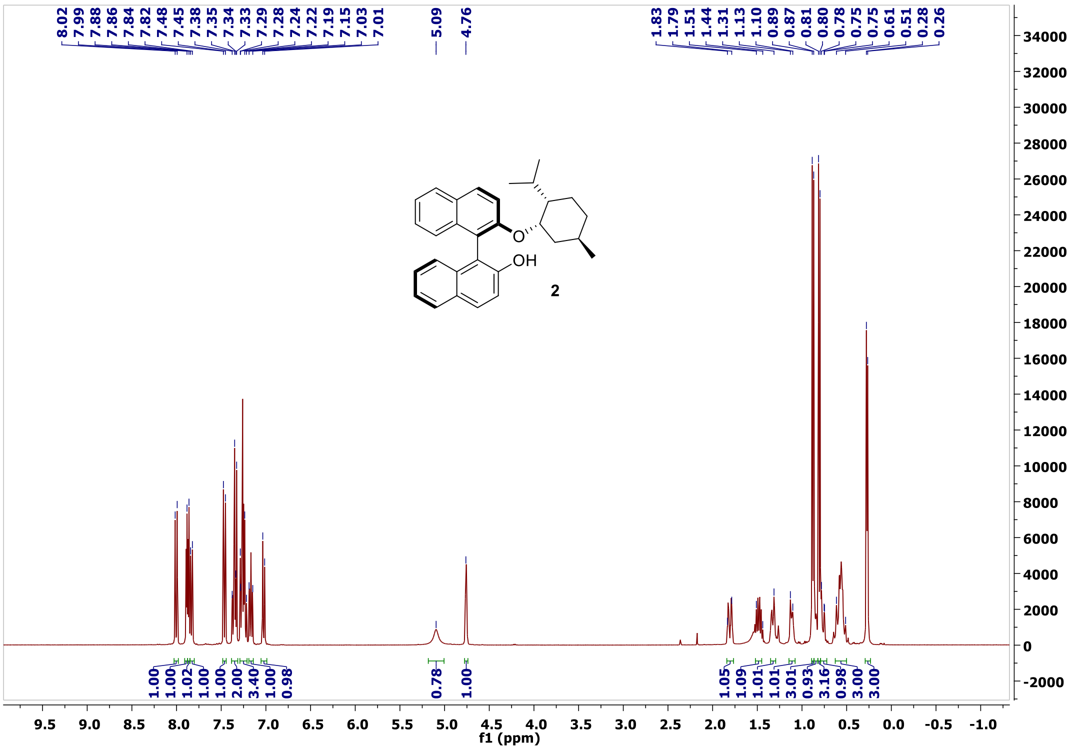

Figure S4. ^1H NMR spectrum of (*R*)-BINOL-(+)-neomenthol (**2**) in $CDCl_3$.

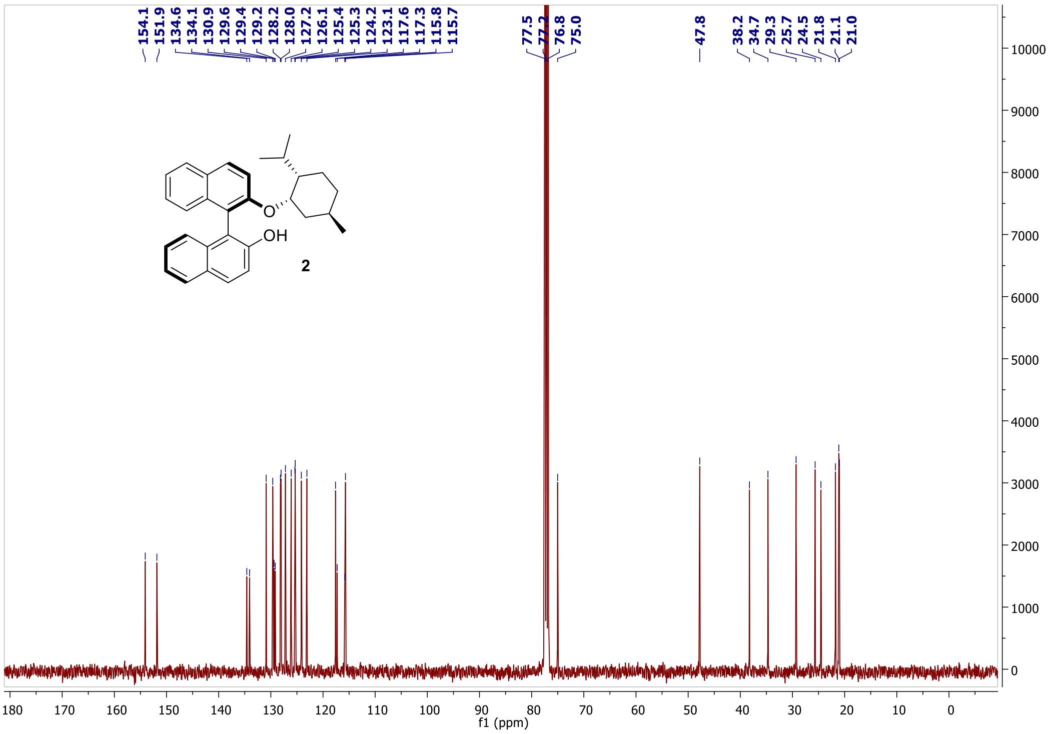

Figure S5. ^{13}C NMR spectrum of (*R*)-BINOL-(+)-neomenthol (**2**) in $CDCl_3$.

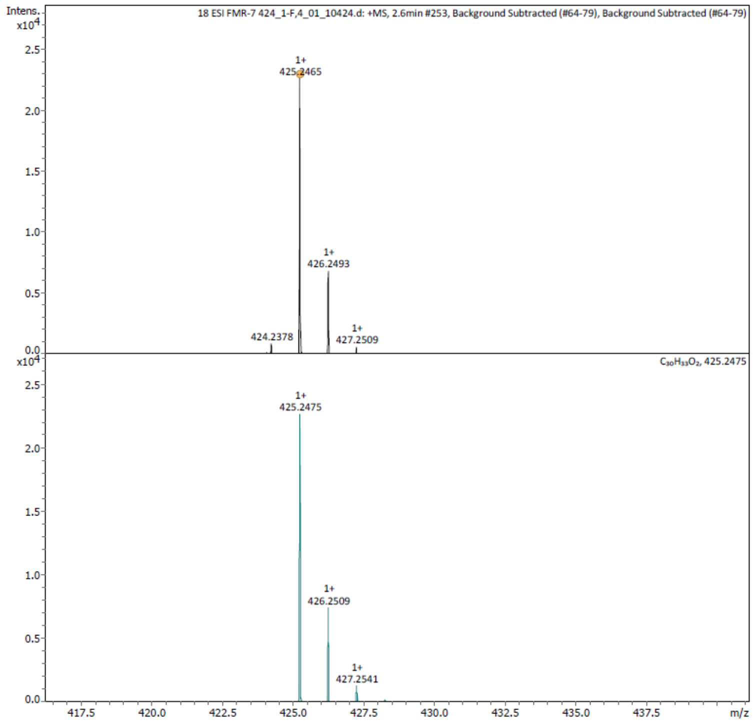

Figure S6. HRMS spectrum of (*R*)-BINOL-(+)-neomenthol (**2**).

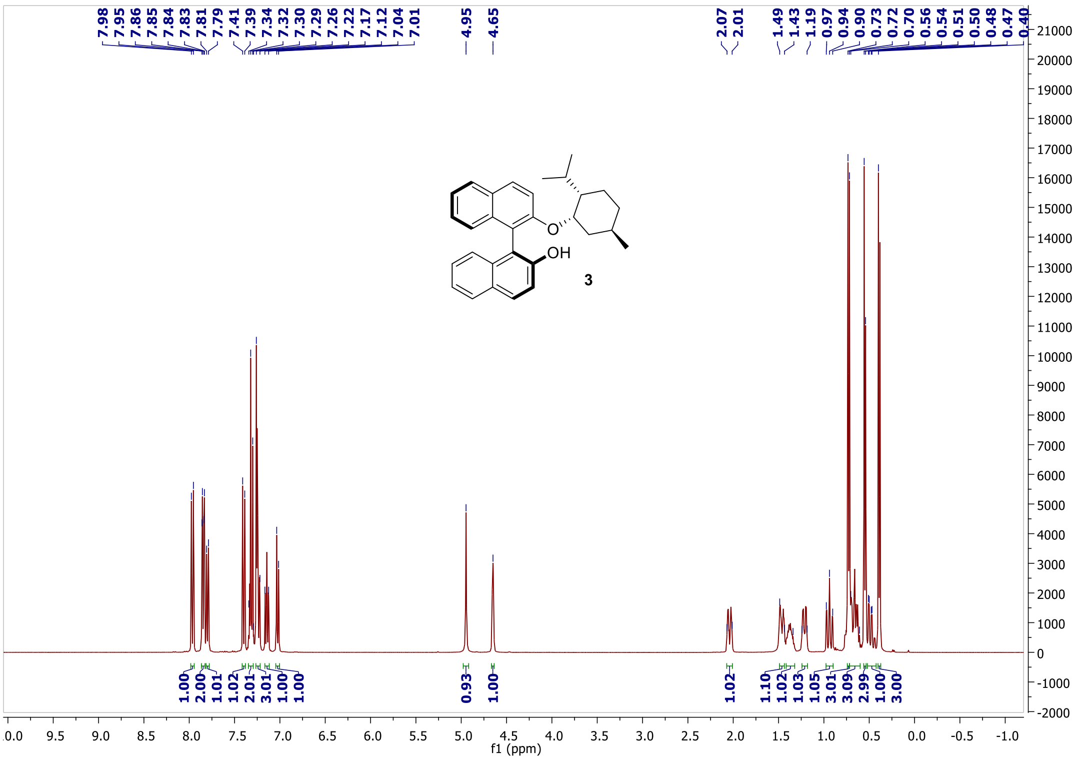

Figure S7. ^{1}H NMR spectrum of (*S*)-BINOL-(+)-neomenthol (**3**) in $CDCl_3$.

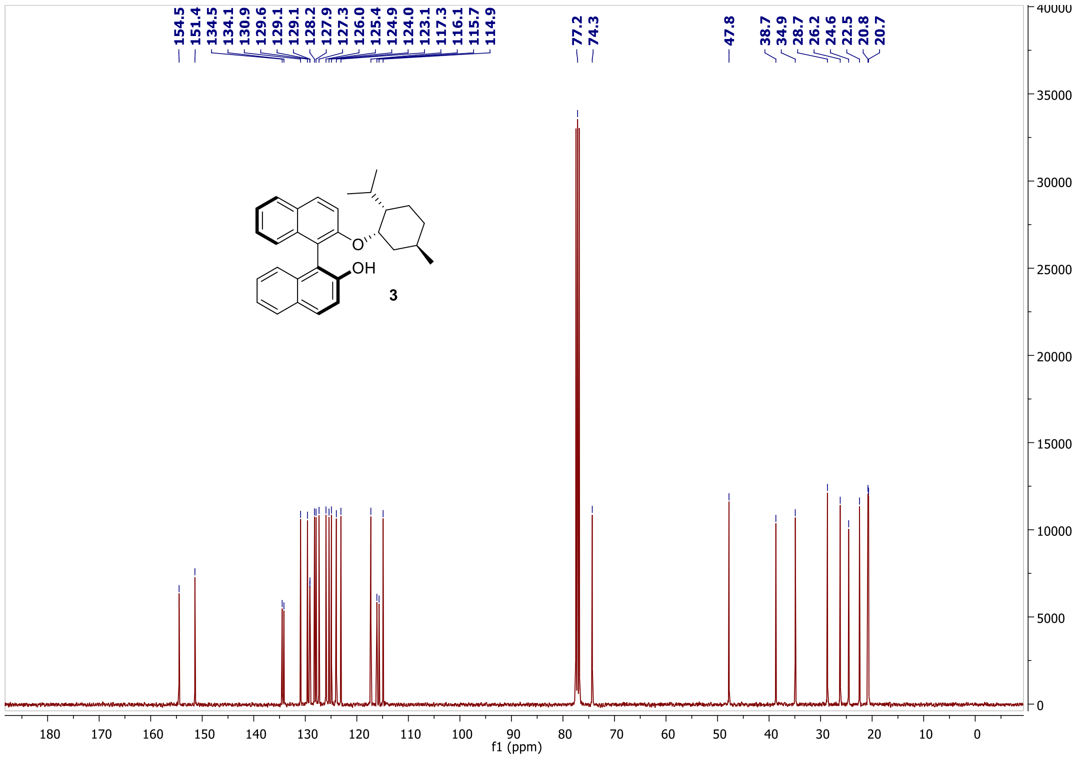

Figure S8. ^{13}C NMR spectrum of (*S*)-BINOL-(+)-neomenthol (**3**) in $CDCl_3$.

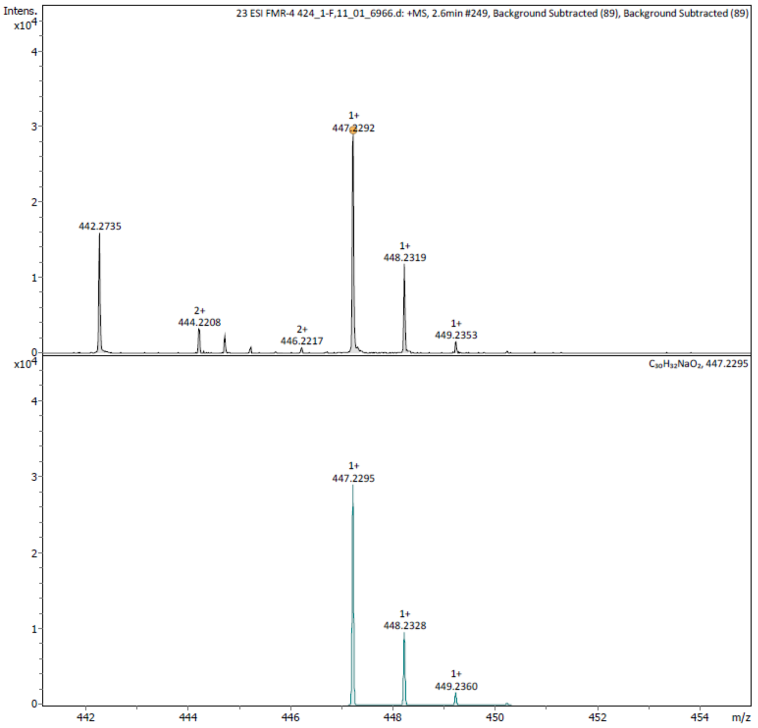

Figure S9. HRMS spectrum of (*S*)-BINOL-(+)-neomenthol (**3**).

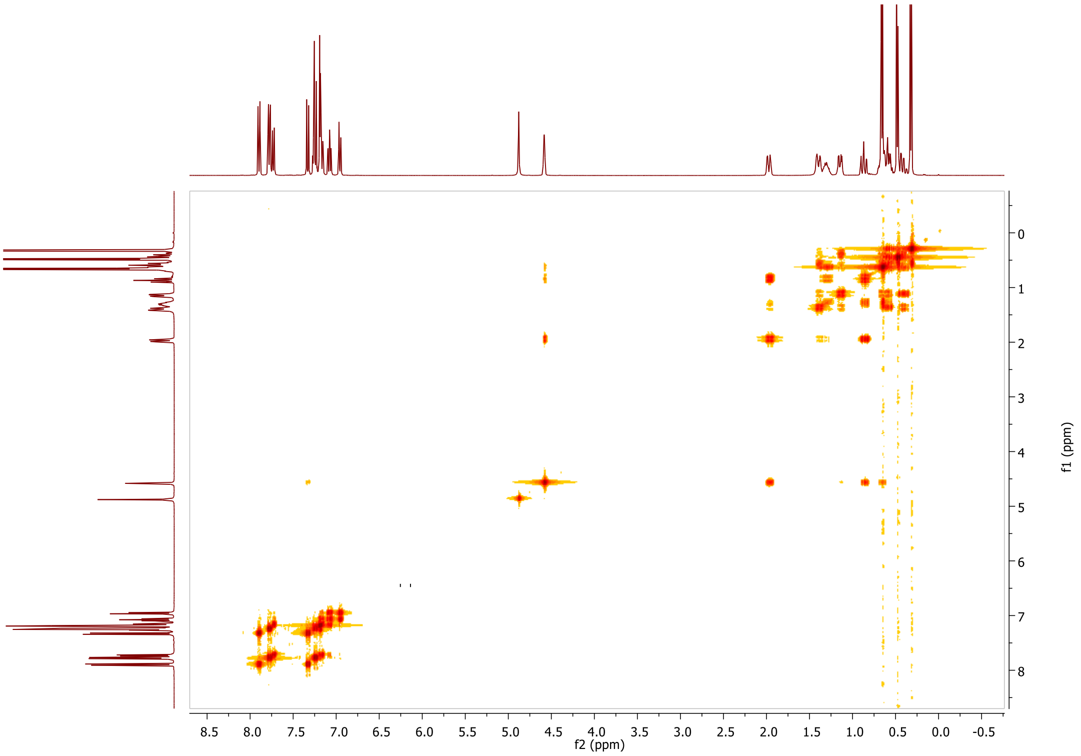

Figure S10. COSY spectrum of (*S*)-BINOL-(+)-neomenthol (**3**) in $CDCl_3$.

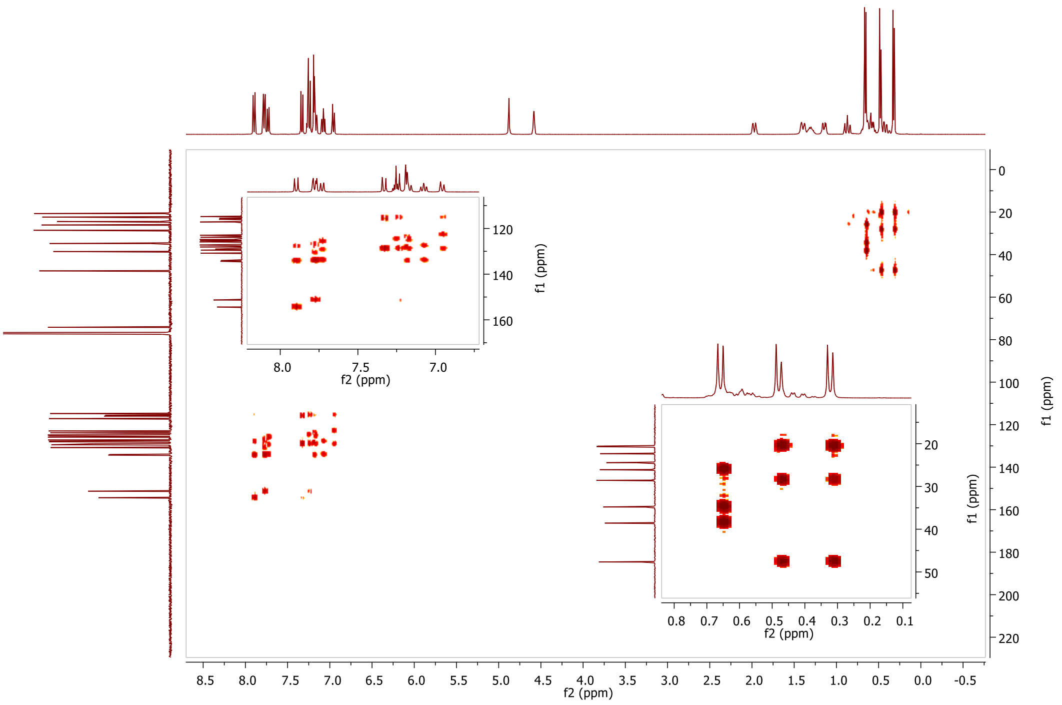

Figure S11. HMBC spectrum of (*S*)-BINOL-(+)-neomenthol (**3**) in $CDCl_3$.

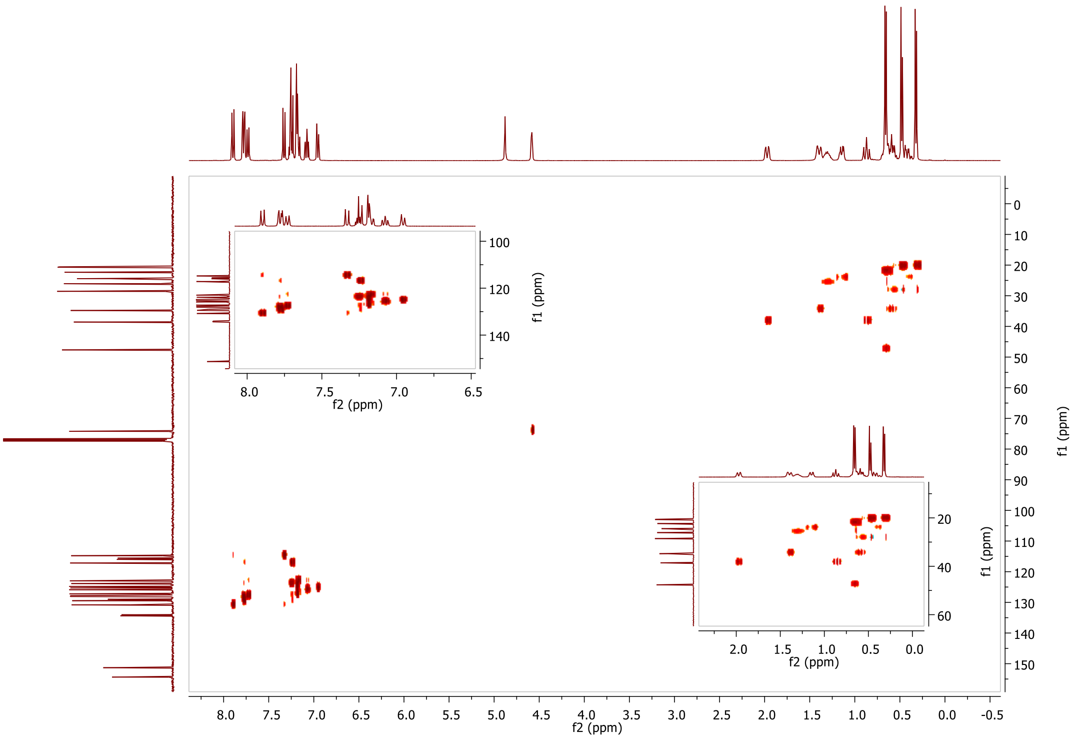

Figure S12. HSQC spectrum of (*S*)-BINOL-(+)-neomenthol (**3**) in $CDCl_3$.

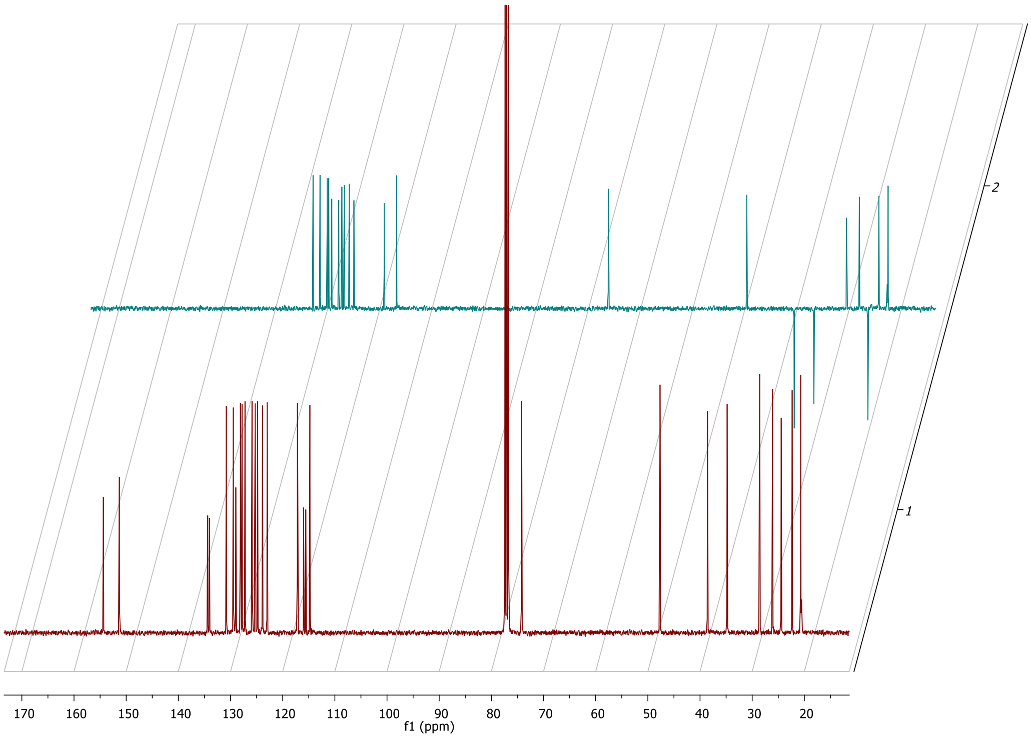

Figure S13. ^{13}C NMR (1) and DEPT 135 (2) spectra of (*S*)-BINOL-(+)-neomenthol (**3**) in $CDCl_3$.

Table S1. ^{1}H and ^{13}C NMR assignments for **3**.

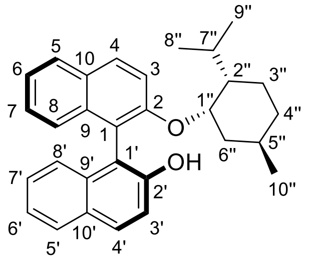

Position	^{1}H NMR / ppm	^{13}C NMR / ppm
1′′	4.65	74.3
2′′	0.69	47.8
3′′	1.22; 0.48	24.6
4′′	1.46; 0.67	34.9
5′′	1.38	26.2
6′′	2.04; 0.94	38.7
7′′	0.64	28.7
8′′	0.55 or 0.40	20.7 or 20.8
9′′	0.55 or 0.40	20.7 or 20.8
10′′	0.72	22.5
OH	4.95	-
1	*	116.1
2	*	154.5
3	7.96	130.9
4	7.40	114.9
5	7.02	125.4
6	7.15	126.0
7	7.22	123.1
8	7.80	127.9
9	*	134.5
10	*	129.1
1′	*	115.7
2′	*	151.4
3′	7.84	128.2
4′	7.31	117.3
5′	7.25	124.9
6′	7.32	124.0
7′	7.25	127.3
8′	7.85	129.6
9′	*	134.1
10′	*	129.1

^{1}H NMR chemical shifts are given as averaged ppm values. For further details, see the full spectra (Figure S7 – Figure S13).
* quaternary C atoms.

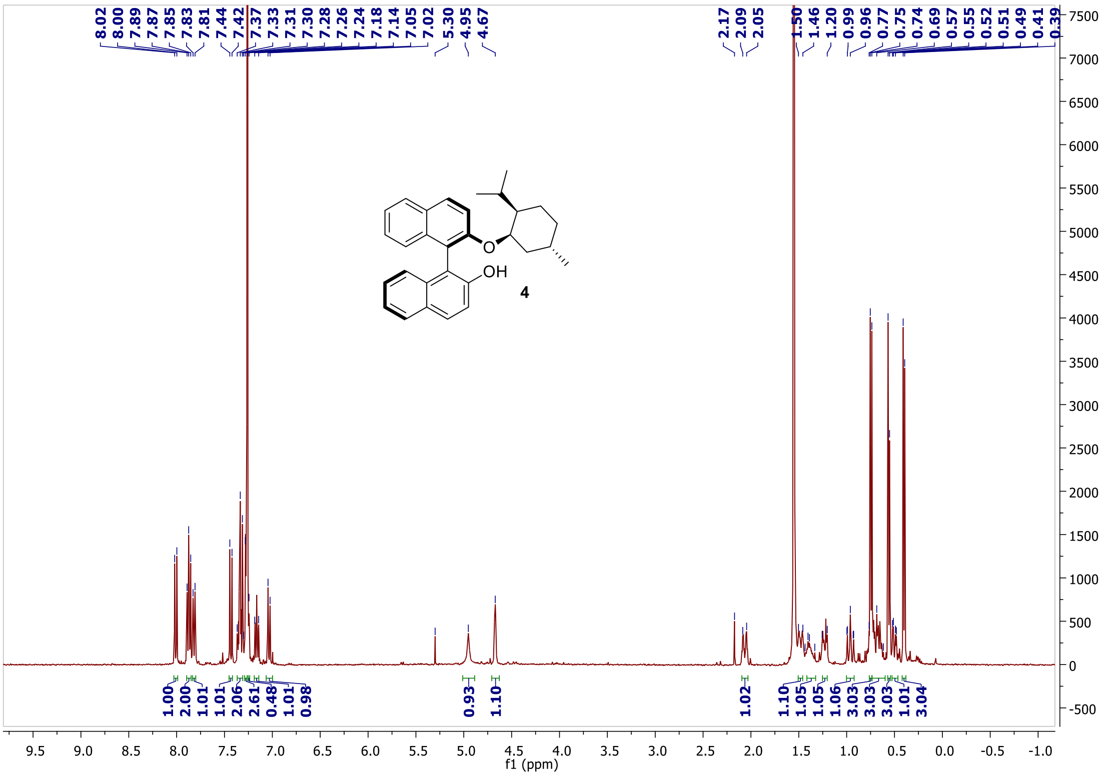

Figure S14. ^{1}H NMR spectrum of (*R*)-BINOL-(−)-neomenthol (**4**) in $CDCl_3$.

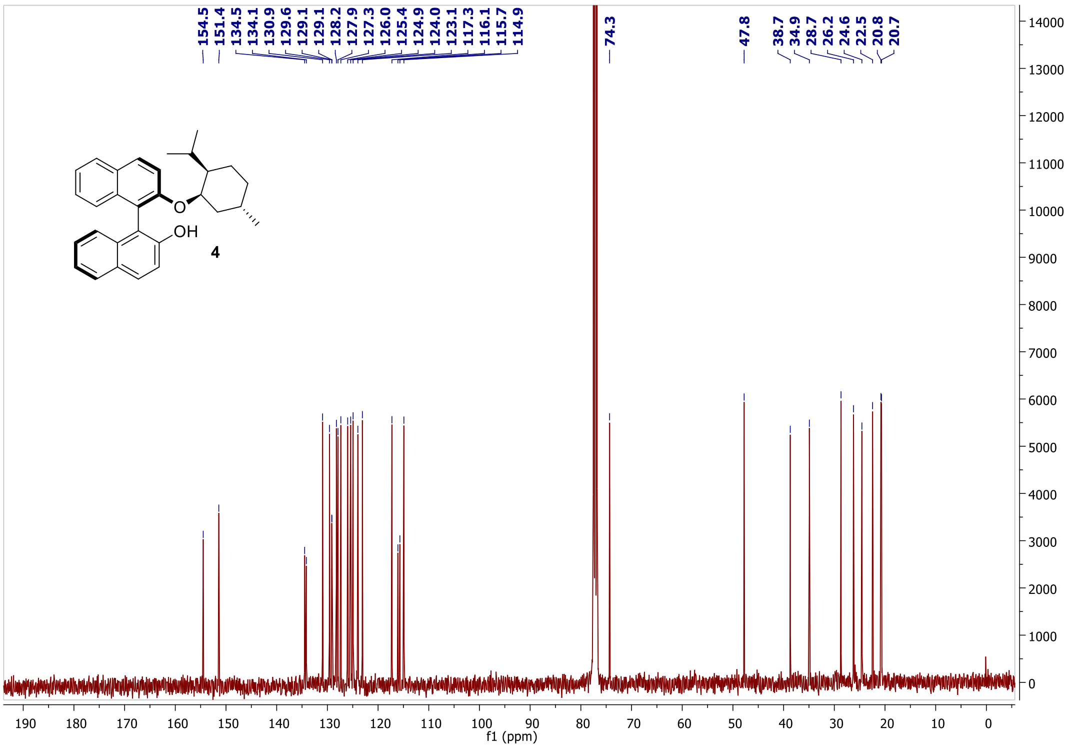

Figure S15. ^{1}H NMR spectrum of (*R*)-BINOL-(−)-neomenthol (**4**) in $CDCl_3$.

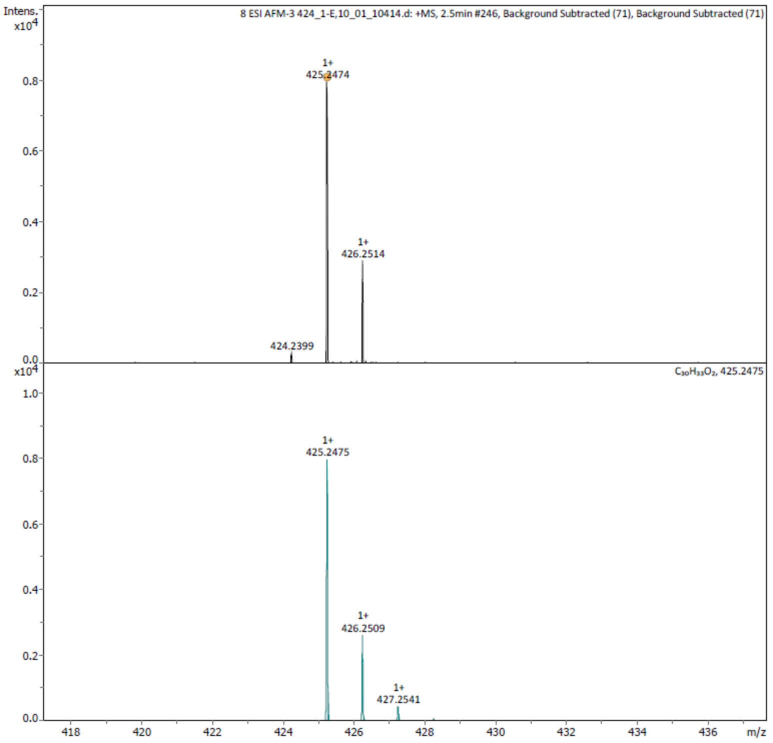

Figure S16. HRMS spectrum of (*R*)-BINOL-(−)-neomenthol (**4**).

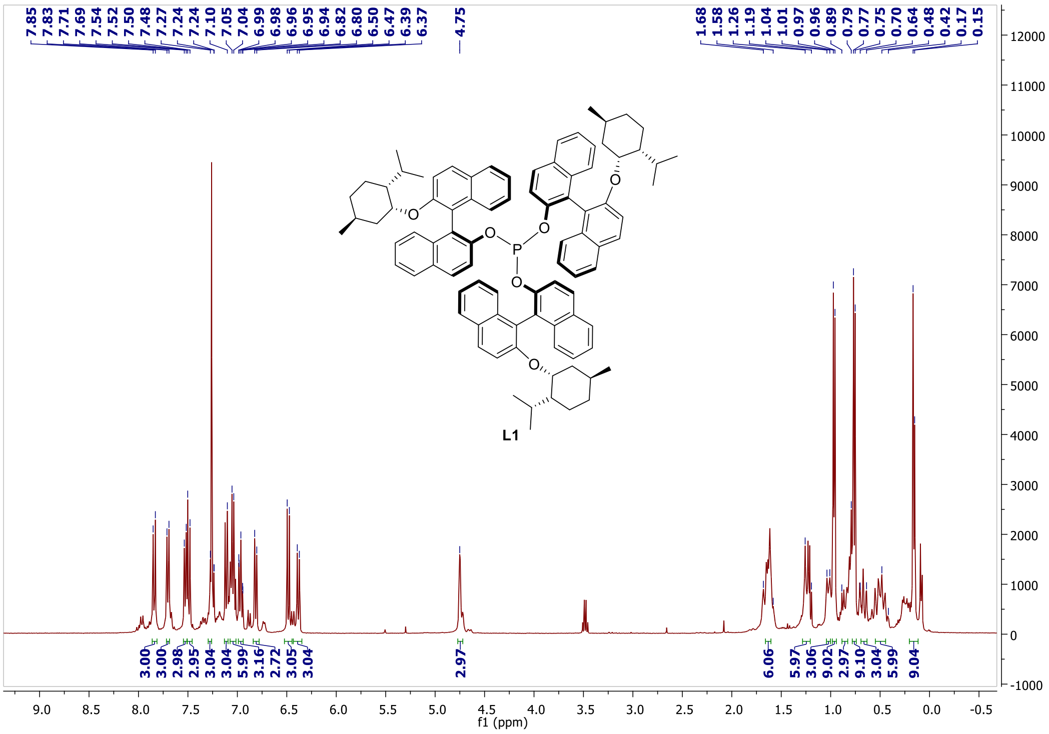

Figure S17. ^{1}H NMR spectrum of **L1** in $CDCl_3$.

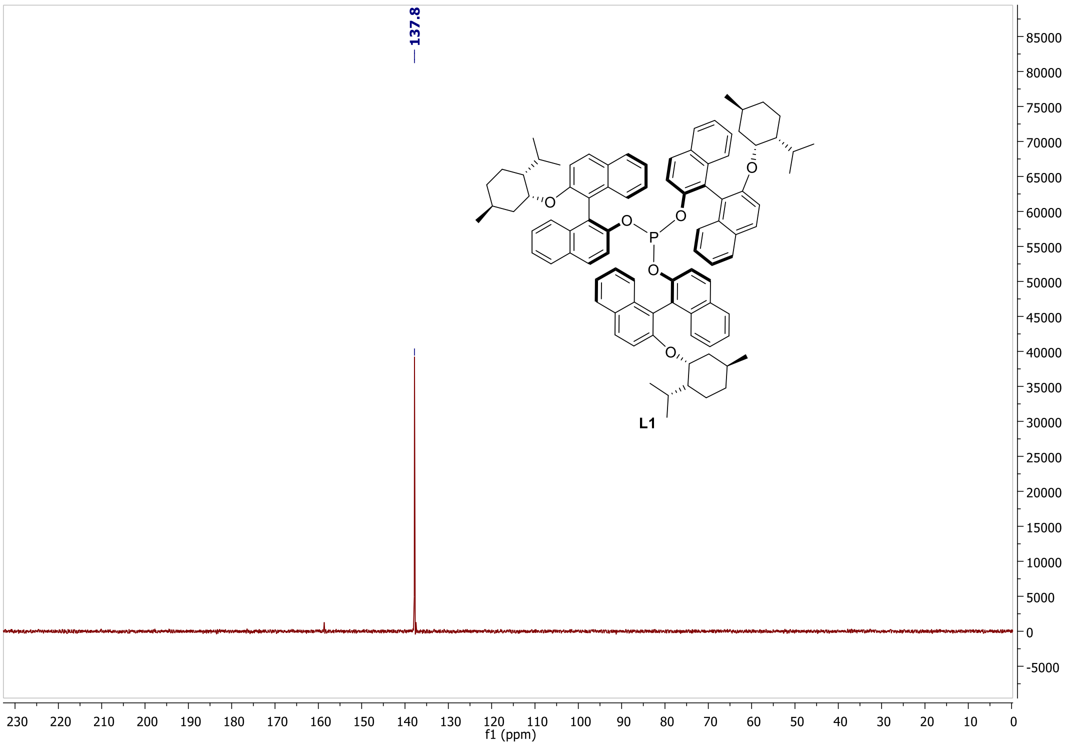

Figure S18. ^{31}P NMR spectrum of **L1** in $CDCl_3$.

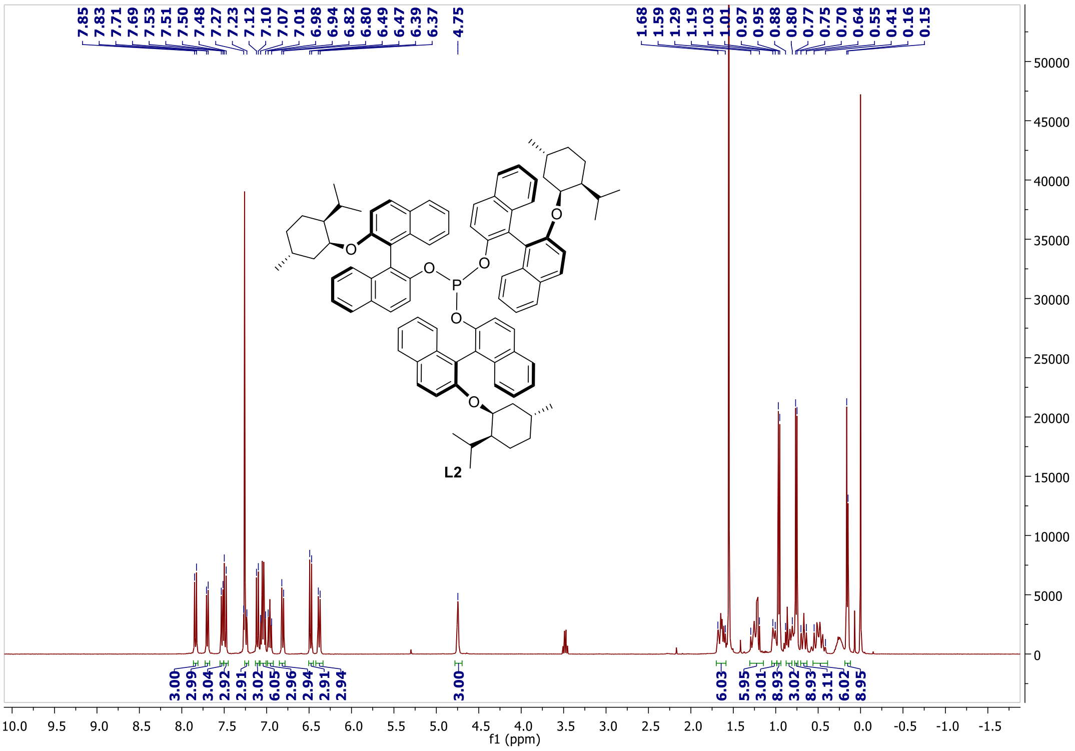

Figure S19. ^{1}H NMR spectrum of **L2** in $CDCl_3$.

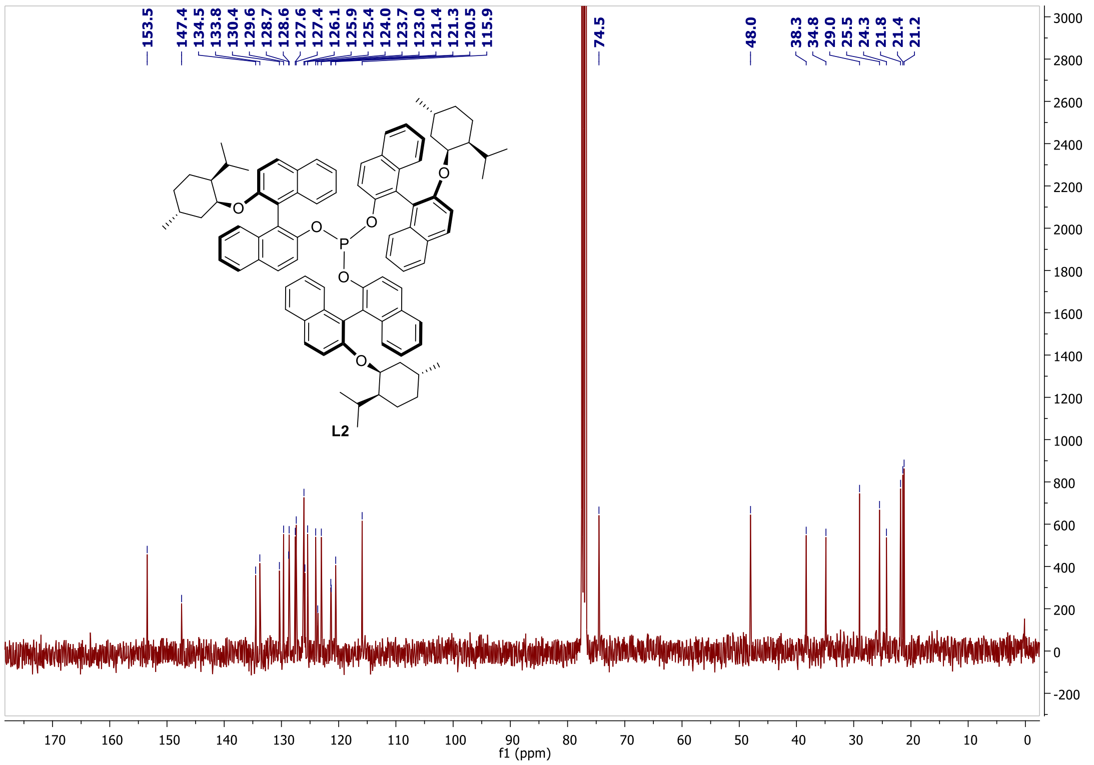

Figure S20. ^{13}C NMR spectrum of **L2** in $CDCl_3$.

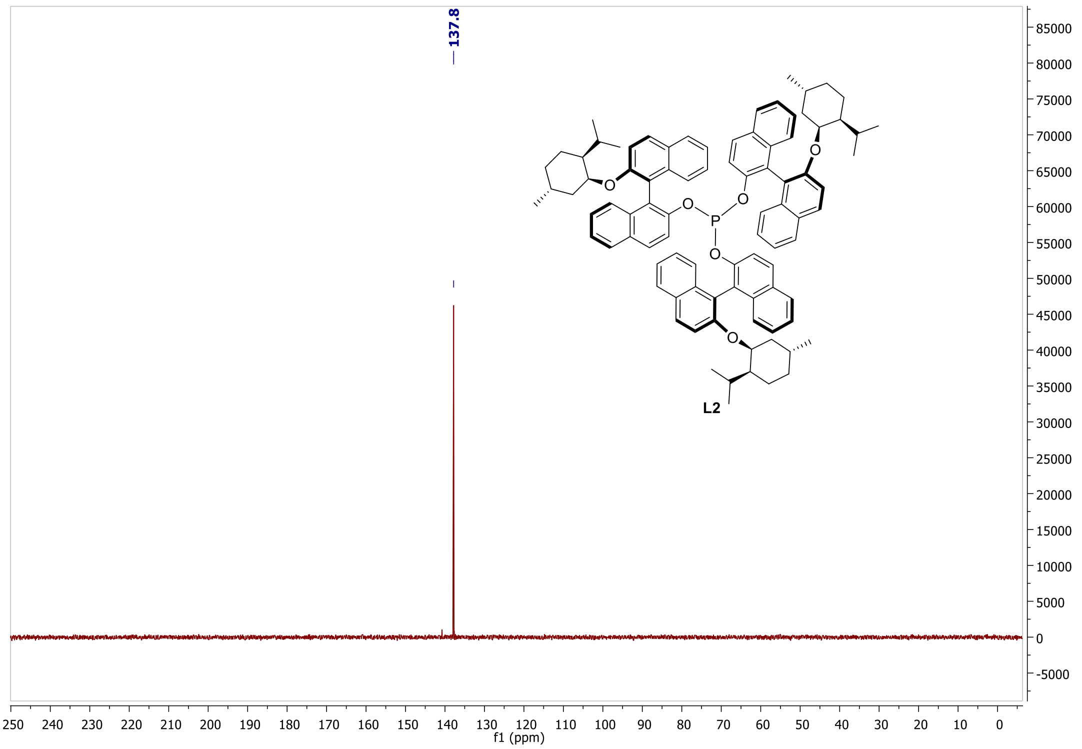

Figure S21. ^{31}P NMR spectrum of **L2** in $CDCl_3$.

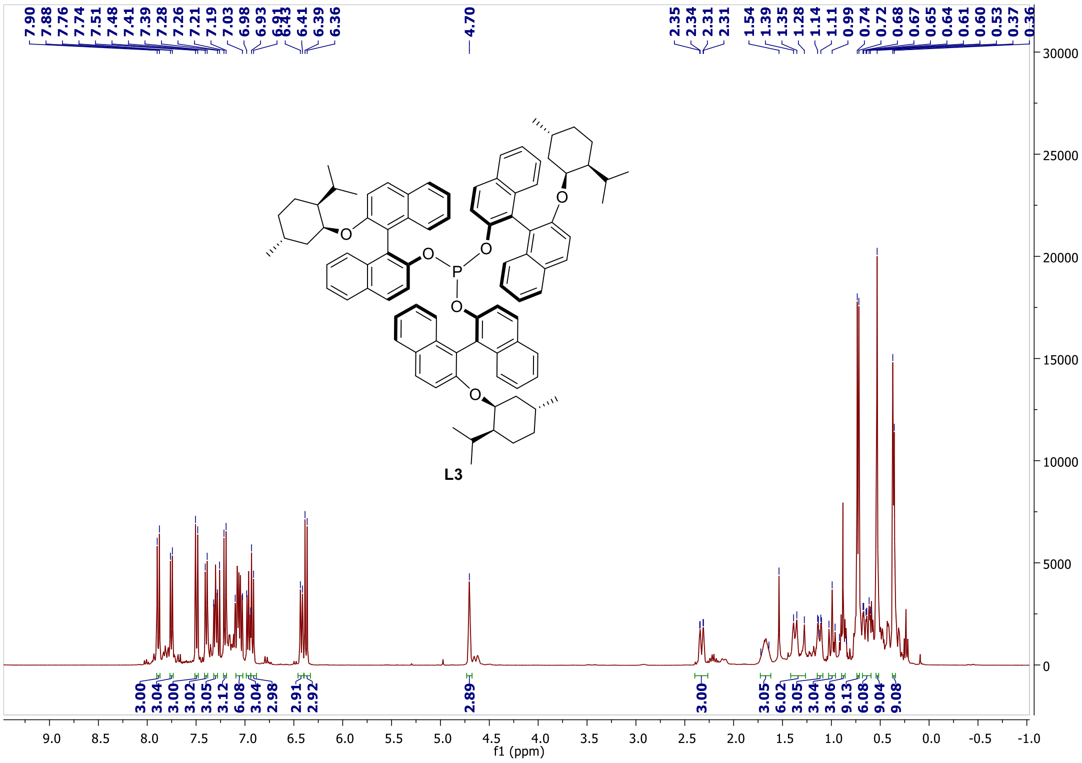

Figure S22. ^{1}H NMR spectrum of **L3** in $CDCl_3$.

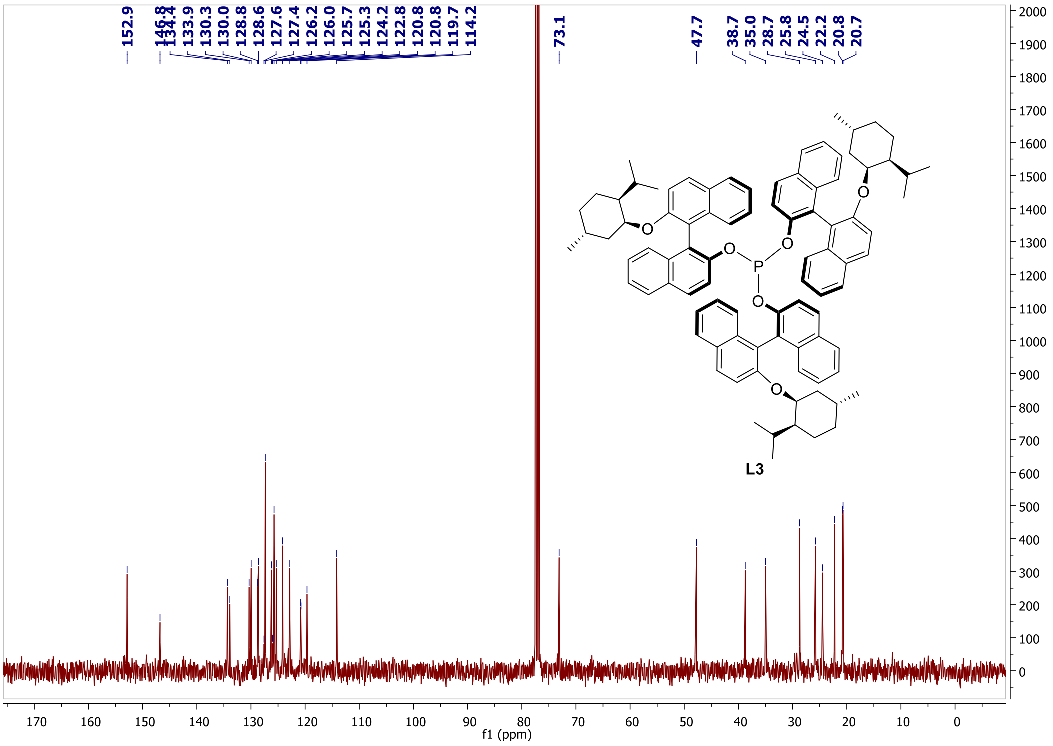

Figure S23. ^{13}C NMR spectrum of **L3** in $CDCl_3$.

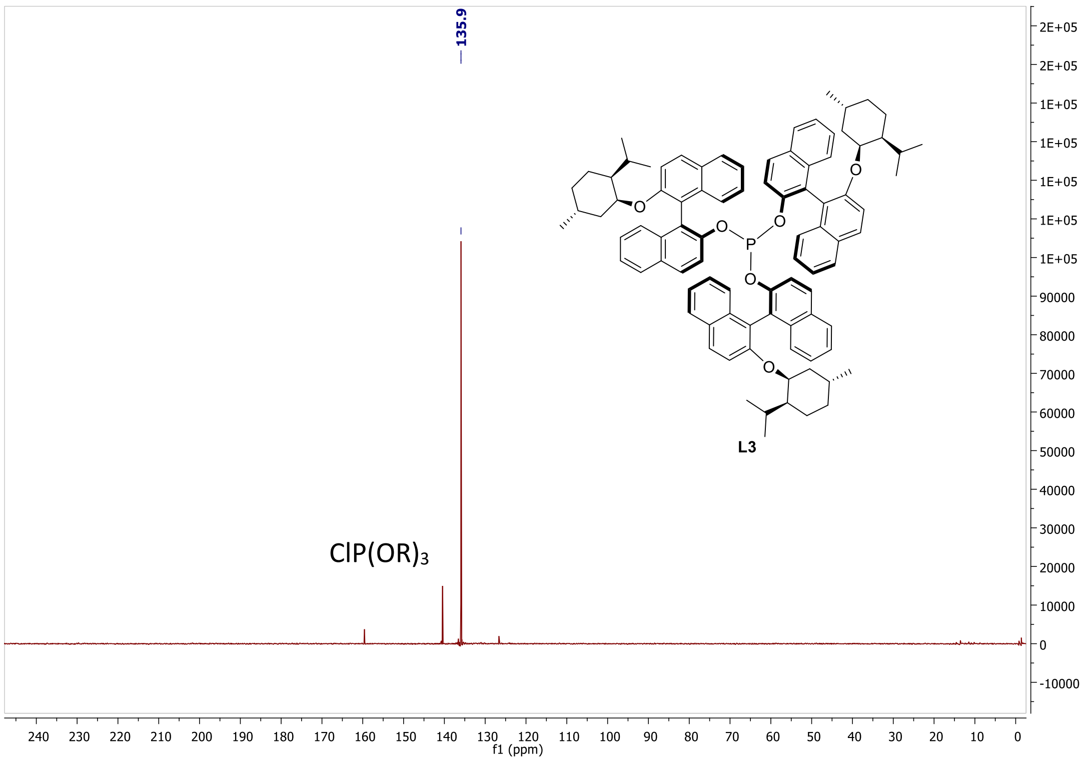

Figure S24. ^{31}P NMR spectrum of **L3** in $CDCl_3$.

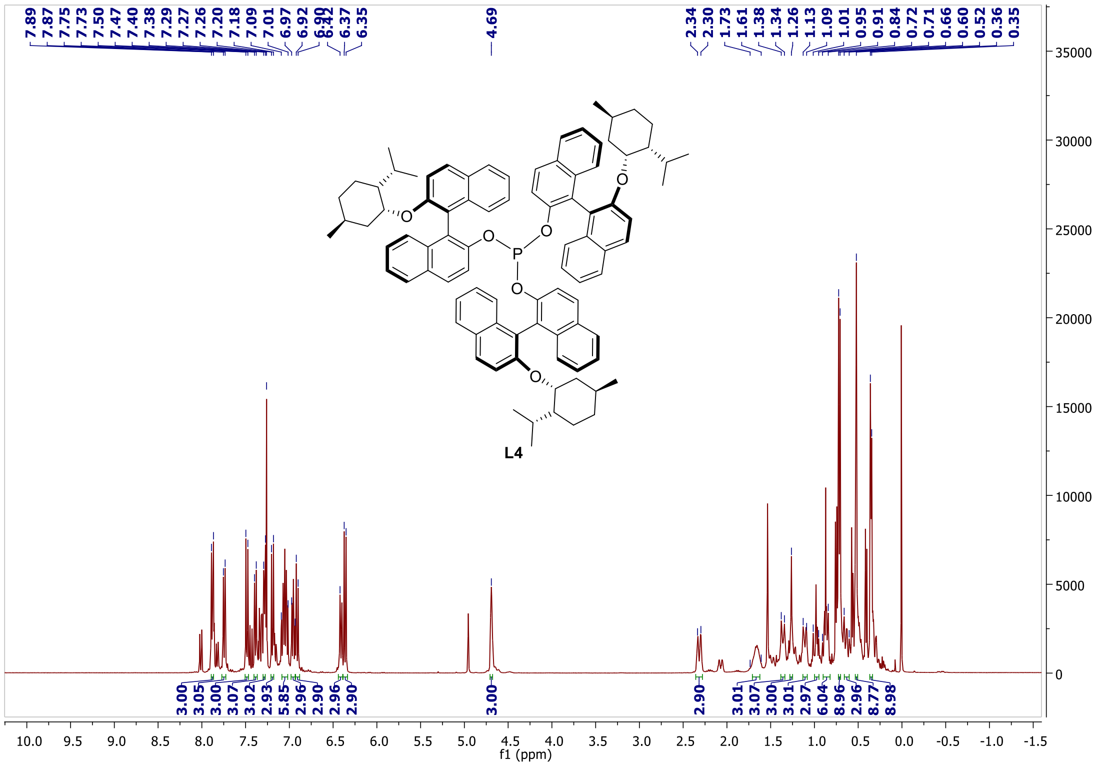

Figure S25. ^{1}H NMR spectrum of **L4** in $CDCl_3$.

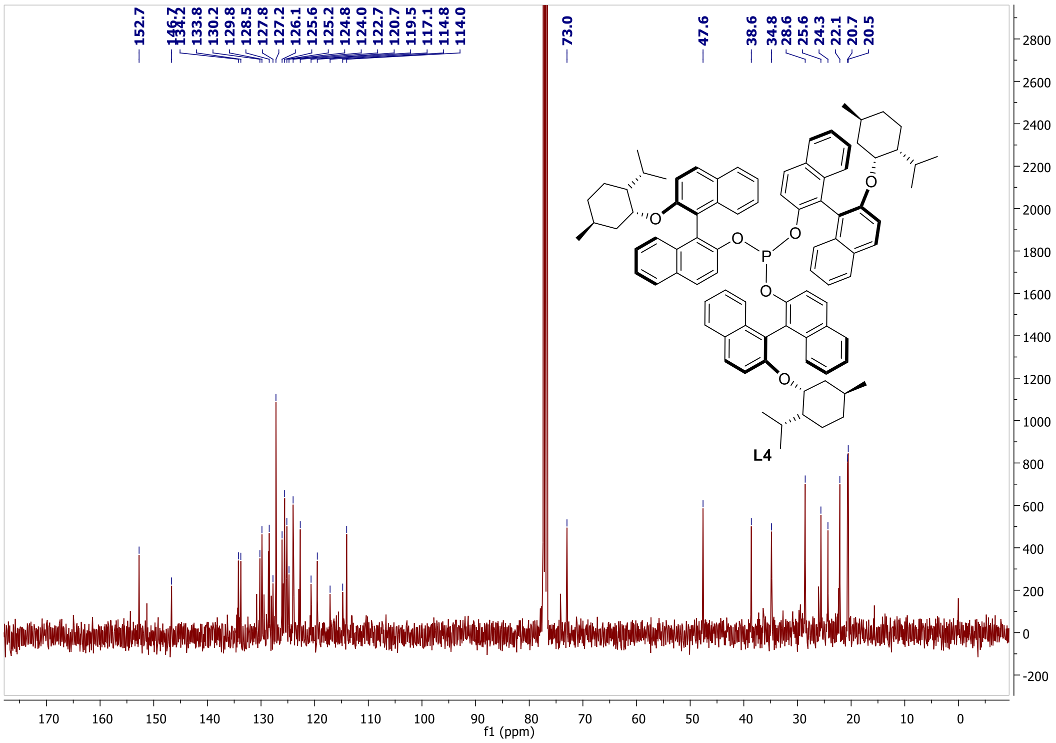

Figure S26. ^{13}C NMR spectrum of **L4** in $CDCl_3$.

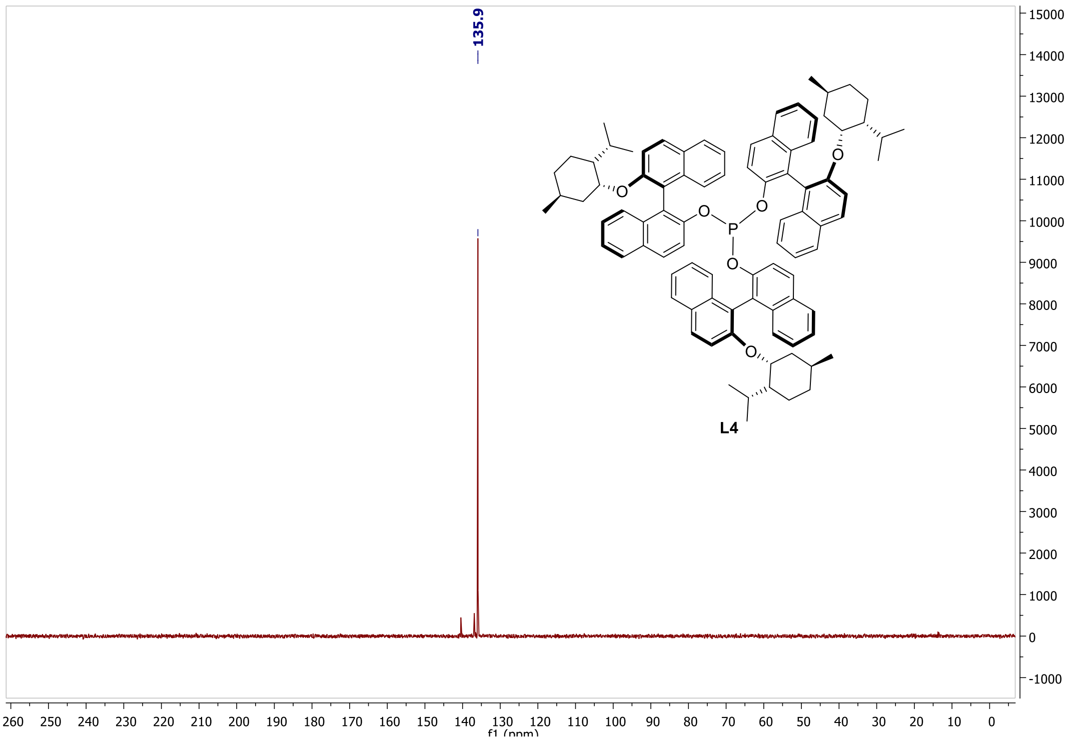

Figure S27. ^{31}P NMR spectrum of **L4** in $CDCl_3$.

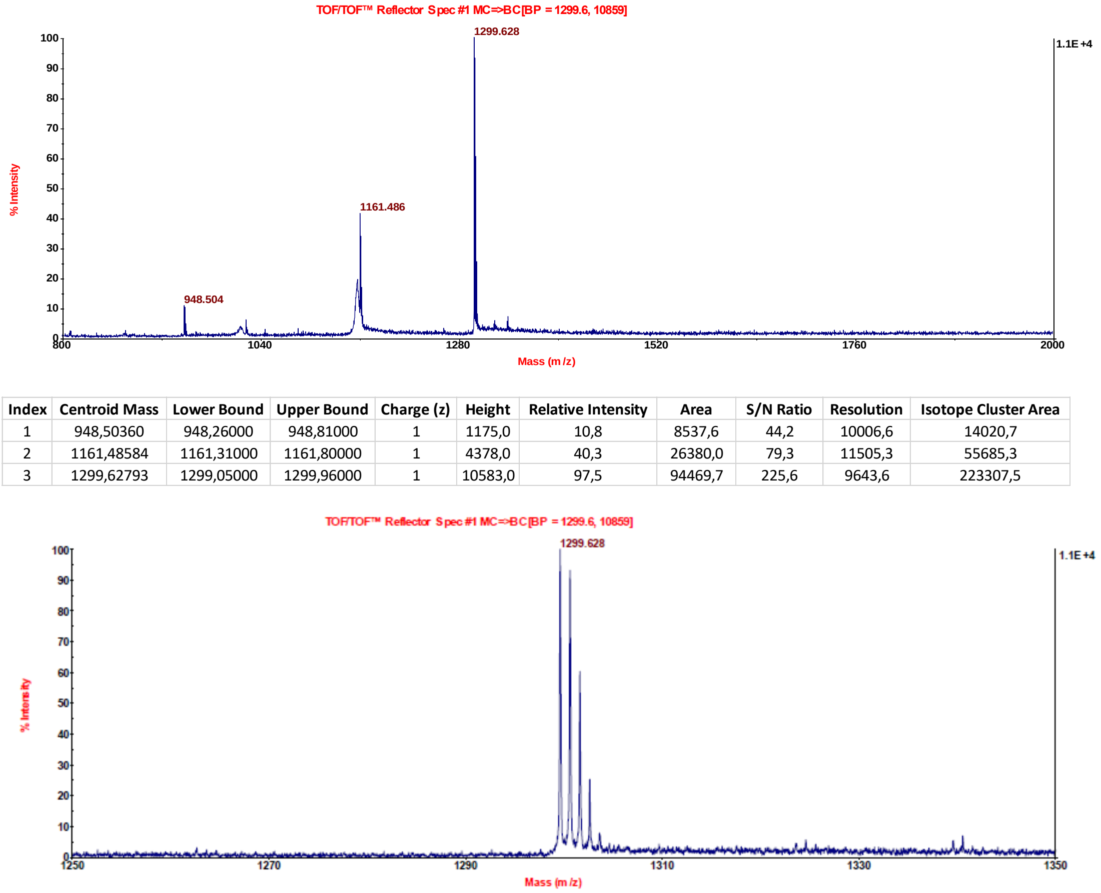

Index	Centroid Mass	Lower Bound	Upper Bound	Charge (z)	Height	Relative Intensity	Area	S/N Ratio	Resolution	Isotope Cluster Area
1	948,50360	948,26000	948,81000	1	1175,0	10,8	8537,6	44,2	10006,6	14020,7
2	1161,48584	1161,31000	1161,80000	1	4378,0	40,3	26380,0	79,3	11505,3	55685,3
3	1299,62793	1299,05000	1299,96000	1	10583,0	97,5	94469,7	225,6	9643,6	223307,5

Figure S28. HRMS (MALDI-TOF) spectrum of **L4**.

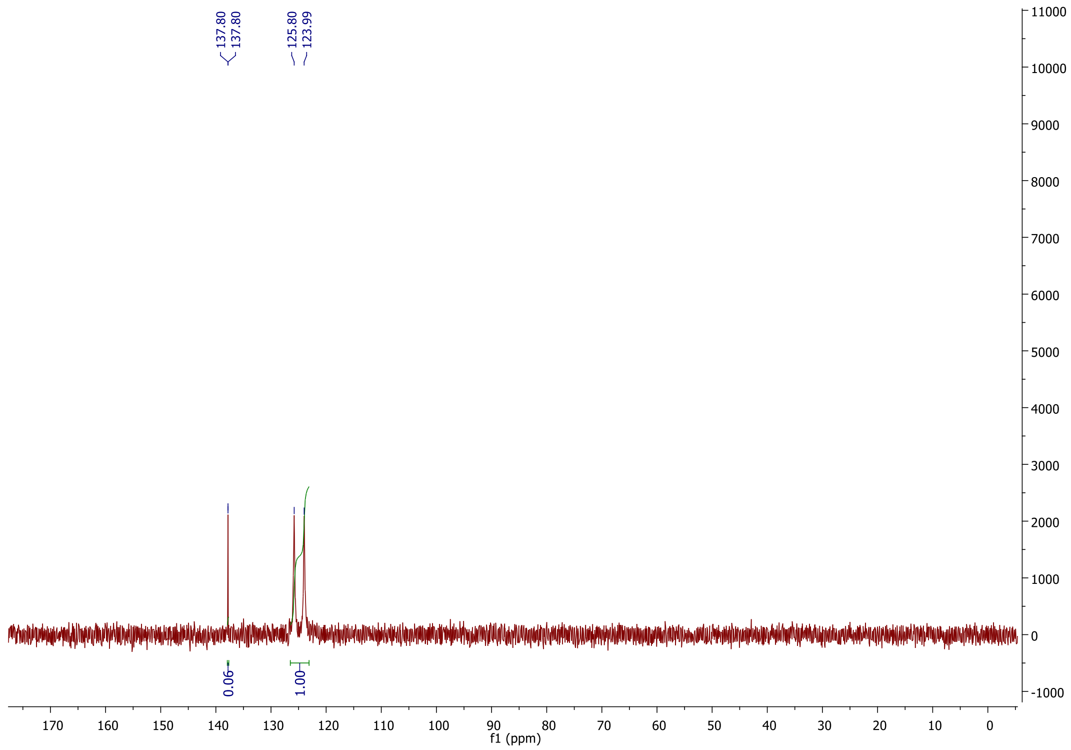

Figure S29. ^{31}P NMR spectra of **L2** + $Rh(CO)_2(acac)$ (1:1), in toluene-d8, at – 3 °C.

2. Crystallographic data

Selected crystallographic data resulting from single crystal x-ray diffraction experiments with samples of compounds **1-4** are included in **Table S2** below. Data collections were carried out at room temperature T=296(2)K, with incident Mo K_α radiation λ=0.71073Å. Full data and refined structural model details can be found in respective CIF files deposited in the CCDC Database with numbers from 2130018 to 2130021.

Table S2. Selected crystallographic data for compounds **1**-**4**.

**Compound**	**1**<br>(*S*)-BINOL-(–)-neomenthol	**2**<br>(*R*)-BINOL-(+)-neomenthol	**3**<br>(*S*)-BINOL-(+)-neomenthol	**4**<br>(*R*)-BINOL-(–)-neomenthol
empirical formula	$C_{30}H_{32}O_2$	$C_{30}H_{32}O_2$	$C_{30}H_{32}O_2$	$C_{30}H_{32}O_2$
molecular mass	424.55	424.55	424.55	424.55
crystal system	monoclinic	triclinic	monoclinic	triclinic
space group	$P2_1$	P1	$P2_1$	P1
a (Å)	8.92(15)	8.554(19)	8.9225(4)	8.669(16)
b (Å)	24.6(4)	10.37(2)	24.5910(11)	10.466(19)
c (Å)	11.03(18)	27.70(6)	11.0687(5)	27.96(6)
α (°)	90	85.20(4)	90	85.23(5)
β (°)	94.0(3)	85.53(3)	93.8380(10)	85.35(4)
γ (°)	90	86.37(3)	90	86.37(4)
unit cell volume	2414(69)	2435(9)	2423.17(19)	2516(9)
Z	4	4	4	4
calculated density (g/cm^3)	1.168	1.158	1.164	1.121
absorption coefficient (mm^{-1})	0.071	0.071	0.071	0.068
F(000)	912	912	912	912
data/restraints/parameters	8528/1/580	17445/3/1158	6939/1/580	29425/3/1169
goodness of fit (S^2)	1.029	1.005	1.065	1.022
